# Molecular Phylogeny and Evolution of the *Tuerkayana* (Decapoda: Brachyura: Gecarcinidae) Genus Based on Whole Mitochondrial Genome Sequences

**DOI:** 10.3390/biology12070974

**Published:** 2023-07-08

**Authors:** Zhengfei Wang, Yuqing Zheng, Xinyue Zhao, Xinyi Xu, Zhiwen Xu, Chong Cui

**Affiliations:** 1 Jiangsu Key Laboratory for Bioresources of Saline Soils, Jiangsu Synthetic Innovation Center for Coastal Bio-Agriculture, Jiangsu Provincial Key Laboratory of Coastal Wetland Bioresources and Environmental Protection, School of Wetlands, Yancheng Teachers University, Yancheng 224001, China; 19895621808@163.com (Y.Z.); 15251075879@163.com (X.Z.); 18082165776@163.com (X.X.); 15722573182@163.com (Z.X.); ccyyznlxsa@163.com (C.C.); 2 Ministry of Education Key Laboratory of Contemporary Anthropology, Department of Anthropology and Human Genetics, School of Life Sciences, Fudan University, Shanghai 200000, China; 3 College of Life Sciences, Henan Normal University, Xinxiang 453007, China

**Keywords:** Decapoda, mitogenome, phylogeny, selective pressure, *Tuerkayana*

## Abstract

**Simple Summary:**

The current classification system of the Brachyura based on complete mitochondrial genomes lacks representative species for many genera and even families. This leads to a confusing and incomplete taxonomy within the Brachyura suborder. The target species in this study belong to Gecarcinidae: *Tuerkayana*, which is an intriguing genus proposed in recent years but lacks complete mitochondrial genome phylogenetic evidence. In our research, we sequenced the complete mitochondrial genomes of four species and compared with the existing dataset of 202 mitochondrial genomes of crabs. Our analyses provide mitochondrial evidence for *Tuerkayana* and shedding light on the division of genera within Gecarcinidae. Furthermore, using the dataset of 206 crab mitochondrial genomes to examine selective pressure in individual codons, and the selective pressure in the *nad6* gene, suggesting its potential role in the evolutionary history of Gecarcinidae.

**Abstract:**

*Tuerkayana* is of particular interest because it has been separated, in recent years, from *Cardisoma* and *Discoplax* but studies of its taxonomic status, especially from a whole mitochondrial genome perspective, have been lacking. In this study, the mitogenomes of four species (*Tuerkayana magnum*, *Tuerkayana rotundum*, *Tuerkayana hirtipes,* and *Tuerkayana celeste*) of *Tuerkayana* are sequenced and contrasted with other species in Brachyura for the first time. The phylogenetic tree of Brachyura, which includes 206 crab species (189 species of Brachyuran and 17 Anomura species) with a complete mitogenome, was constructed to evaluate the phylogenetic position of *Tuerkayana* and Gecarcinidae within Brachyuran, and explore the monophyly of Gecarcinidae. Furthermore, two single gene trees based on *cox1* and *16SrRNA* separately within interspecies of Gecarcinidae were reconstructed, providing molecular evidence for *Tuerkayana* and further clarifying the division of genera in Gecarcinidae. Based on the mitogenome dataset of 206 crabs, the branch-site model was utilized to explore selective pressure in individual codons with CodeML. The strong selective pressure shown in *nad6* indicates that it may have played a significant role in the evolution of Gecarcinidae.

## 1. Introduction

Due to its rapid evolutionary rate, maternal inheritance, small size, conserved gene content, and relatively easy acquisition, the mitochondrial genome (mitogenome) plays a key role in evolutionary studies [1]. The complete mitochondrial sequence contains more biological information than single gene molecular markers and can effectively avoid the interference of nuclear pseudogenes [2]. The mitogenomes within metazoan are generally 14–20 kb in length, comprising two ribosomal RNA genes (*12SrRNA* and *16SrRNA*), 22 transfer RNA genes (tRNAs), 13 protein-coding genes (PCGs), and 1 control region (CR, or D-loop region). Complete mitogenome sequences are usually utilized in phylogenetic research, comparative genomics, population genetics, and molecular evolution [3]. The important information provided by mitogenome data regarding sequences and genomic rearrangements, which are generally complex and diverse, partly reflects the evolution of animals [4].

At present, Gecarcinidae is considered to have 7 genera (*Cardisoma*, *Discoplax*, *Gecarcinus*, *Gecarcoidea*, *Johngarthia*, *Epigrapsus*, and *Tuerkayana*) and 26 species. The main object of our study, *Tuerkayana*, is a newly established genus in recent years [5], which includes five species separated from *Discoplax* (*Tuerkayana celeste* and *Tuerkayana magnum*), *Cardisoma* (*Tuerkayana rotundum*, *Tuerkayana hirtipes*), and *Tuerkayana latens*. However, the taxonomic status of the genus, especially from a whole mitochondrial genome perspective, has been lacking. Mitogenome information in Gecarcinidae is available for only four sequenced species belonging to genera *Cardisoma* (*Cardisoma armatum* and *Cardisoma carnifex*) and *Gecarcoidea* (*Gecarcoidea lalandii* and *Gecarcoidea natalis*), and other genera still lack representative species. This situation, with the phylogeny not yet resolved and requiring further investigation, has caused difficulties in confirming the monophyly of *Tuerkayana*.

In addition, beyond that, we have taken note of the adaptability that may be manifested by mitochondrial selection pressure. Gecarcinidae crabs, a semi-terrestrial family in the Brachyura group, are generally distributed in coastal burrows, rock crevices, and coastal thickets [6,7]. Adult crabs of Gecarcinidae are generally terrestrial, but they seasonally spawn in seawater, and their larva grows in water prior to maturity [8]. For example, the Christmas Island red crab (*Gecarcoidea natalis*) migrates yearly to the coast for breeding [9]. On account of the terrestrial characteristics of Gecarcinidae, many studies have been published regarding its morphological structure, reproduction, tolerance level, feeding habit, and other aspects [9,10,11,12,13]. Evidence of periodic transitions from marine to terrestrial environments suggests land crabs could be the result of single adaptive radiation from a marine ancestor which invaded terrestrial habitats [14]. Land crabs may therefore be hypoxic-adapted [15]. Oxygen plays a central role in the mitochondrial respiratory chain during the OXPHOS process, acting as the terminal electron acceptor of the electron transport chain (ETC) and enabling the synthesis of adenosine triphosphate (ATP) [16]. Accompanying inflation and a posterior narrowing of the carapace, the reduction of gill filaments, and the occurrence of branchiostegal lungs [10,17], hypoxia may have imposed important selective constraints on the evolution of the mitogenome in terrestrial crabs.

*T. magnum*, *T. rotundum*, *T. hirtipes,* and *T. celeste* are distributed in the Indo–West Pacific region and are highly terrestrial, usually settling in the vegetation above shorelines and shallow burrows near freshwater. In this study, the complete mitogenome sequences of four species in *Tuerkayana* were determined and analyzed for the first time. To ensure the conclusions we reached were reliable, we used a variety of existing complete mitogenomes in this study and compared their molecular features with *Tuerkayana*. The phylogenetic tree of Brachyuran was reconstructed, based on the sequences of 13 PCGs from 206 species, to analyze the selection pressure of 13 PCGs. The reconstructed phylogenetic tree of Brachyuran enables the division of genera in the family Gecarcinidae to be better understood. The tree was constructed to provide evidence for the establishment of *Tuerkayana* at the mitochondrial level. In addition, based on the results of the selection pressure analysis, the influence of the terrestrial adaptation of Gecarcinidae in the mitochondrial genes was investigated.

## 2. Materials and Methods

### 2.1. Samples and the Extraction of DNA

Two samples of each of the four species (*T. magnum*, *T. rotundum*, *T. hirtipes*, and *T. celeste*) were purchased from Indonesia. The samples entered China in accordance with the Animal Quarantine Law of the People’s Republic of China and the Animal Quarantine Control Measures. One of the samples was stored as a copy in the freezer at −80 °C. The other was dissected for muscle samples, which were flash-frozen under liquid nitrogen and stored in the freezer at −80 °C in Yancheng Teachers University for extracting the total genomic DNA as per the manufacturer’s instructions of the Aidlab Genomic DNA Extraction Kit (Aidlab Biotech, Beijing, China); 1% agarose gel electrophoresis (0.2 g agarose, 20 mL 1 × TAE, and 2 μL EB) was used to evaluate the quality of the extracted DNA samples, which were stored at −20 °C for further polymerase chain reaction (PCR) if they were of sufficiently high quality.

### 2.2. PCR Amplification and Sequencing

The conserved sequences were amplified by PCR using the universal primer *cox1* and *16SrRNA* (Table S2) [4,18]. PCR was undertaken with a 25 μL mixed system (12.5 μL of 2 × F8 PCR MasterMix; 0.5 μL of forward primer and reverse primer, respectively; 1.5 μL DNA template; and 10 μL ddH_2_O) on a DNA amplification apparatus (ABI9700). The thermocycling profile was operated in 94 °C for 5 min, followed by 34 cycles, 95 °C for 30 s, 53–59 °C for 30 s, 72 °C for 30 s, followed by a 10 min extension at 72 °C. Subsequently, the quantity and the quality were assessed with 1% agarose gel electrophoresis.

The complete mitogenomes were sequenced using next-generation sequencing. *T. magnum* was sequenced with Shanghai Origingene Bio-pharm Technology Co. Ltd., Shanghai, China (Illumina HisSeq 4000), and the other three species (*T. celeste*, *T. rotundum*, and *T. hirtipes*) were sequenced with majorbio Bio-Pharm Technology Co. Ltd., Shanghai, China (Illumina HisSeq 6000). To ensure consistency between species names and sequences, the assembled mitochondrial genes were identified by checking the *cox1* barcode sequence on NCBI with NCBI’s Basic Local Alignment Search Tool (BLAST) search function for nucleotide sequences (BLASTn; available at: https://www.ncbi.nlm.nih.gov/; accessed on 7 November 2021) [19]. BLASTn searches, using putative orthologous sequences from other crab species obtained from NCBI, was used to query the non-redundant nucleotide (nr/nt) sequence database in order to discover annotated putative orthologue genes. Clean data were assembled from scratch without sequencing adapters using NOVOPlasty [20]. To evaluate the single-base accuracy of the assembled mitogenome (assembled mitochondrial genome) with two confirmed sequences, it was contrasted with Sanger sequencing. The complete mitogenome was uploaded to GenBank (accession number ON990061, OQ948153-OQ948155, available at NCBI).

### 2.3. Sequence Analysis and Gene Annotation

The PCGs were identified using NCBI and MITOS Web Server [20] (http://mitos2.bioinf.unileipzig.de/index.py; accessed on 20 March 2022) to analyze the mitogenome code of invertebrates. Codon usage was assessed with MEGA 7.0 [21] based on PCGs with incomplete stop codons removed. The secondary structures of tRNAs were determined with MITOS Web Server and tRNAscan-SE Web Server (http://lowelab.ucsc.edu/tRNAscan-SE/; accessed on 23 March 2022) [22]. Moreover, MEGA was applied to confirm compositional skews and calculated through the following formulas: AT-skew = (A − T)/(A + T) and GC-skew = (G − C)/(G + C) [23], and calculated genetic distance analysis (p-distance) [24].

The gene maps of the mitogenomes of four species were generated and visualized with Organellar Genome DRAW (https://chlorobox.mpimp-golm.mpg.de/OGDraw.html; accessed on 25 March 2022) [25]. In addition, the RNAfold WebServer (http://rna.tbi.univie.ac.at/; accessed on 2 May 2022) was used to predict secondary structures of rRNA and CR, and the Tandem Repeats Finder server (http://tandem.bu.edu/trf/trf.html; accessed on 10 May 2022) was used to identify tandem repeats of the control region.

### 2.4. Phylogenetic Analysis and Gene Rearrangement

In conformity with the phylogenetic trees conducted with the 13 PCGs of all crabs downloaded from GenBank, the taxonomic status of *Tuerkayana* was confirmed. Except for the newly sequenced mitogenomes, the complete mitogenomes of 189 Brachyuran species belonged to 39 families and 17 Anomura species as the outgroup (Table S1) were included. MEGA7.0 [26] was used to align the 13 PCGs of 206 mitogenomes with the default nucleotide and amino acid sequences set in MUSCLE 3.8 [27]. The aligned PCGs were then concatenated into datasets. Before reconstruction, we used DAMBE [28] to detect the nucleotide substitution saturation rate of the aligned PCGs. The phylogenetic tree was reconstructed by the concatenated set of the nucleotide sequence and amino acid sequences, respectively. MtArt + I + G, which was selected by ProtTest, was determined as the best model for analyzing nucleotide sequences [29]. Using Bayesian Inference (BI) and Maximum Likelihood (ML) methods, four phylogenetic trees were constructed with MrBayes v3.2.6 [30] and IQ-TREE [31], respectively. ML bootstrap values (BS) ≥ 75% and Bayesian posterior probabilities (BPP) ≥ 0.95 were considered significant. In the analysis of BI, we made two simultaneous 10,000,000 generation runs to encourage swapping among the Markov chain Monte Carlo (MCMC) chains and sampled trees every 1000 generations. The convergence of sampled parameters and potential autocorrelation (the effective sampling size of all parameters > 200) were investigated by Tracer 1.6 (http://tree.bio.ed.ac.uk/software/tracer; accessed on 5 July 2022), and the average standard deviation of split frequencies was inspected between runs (<0.01). The first 25% of trees were in the burn-in stage. These were removed, and the BPPs could be obtained from the 50% majority rule consensus of the postburn-in trees sampled at stationarity. Subsequently, the final phylogenetic trees were visualized and edited using FigTreev1.4.2.

Two single gene phylogenetic trees (Table S1) were constructed based on the *cox1* and *16SrRNA* from 11 Gecarcinidae species (including all *Discoplax*, *Tuerkayana,* and *Cardisoma* species) and a Menippidae species (*Pseudocarcinus gigas*), respectively. Similar to the above phylogenetic trees of Brachyura, the datasets of nucleotide sequences were used to construct the trees using BI and ML.

Regarding gene rearrangement, MITOS [20] and NCBI [32] were used to re-annotate all the different mitogenomes to ensure the accuracy, consistency, and comparability of the studied species. If differences in the mitochondrial genome are found, they are repaired artificially. In order to examine the underlying evolutionary mechanisms, the CREx Web Server was used. The Common Interval Rearrangement Explorer (CREx) [33] was used to flexibly calculate complex rearrangement mechanisms by means of mathematical models. Under normal circumstances, a single transformation was frequently utilized to determine the most economical mechanism from a variety of possibilities.

### 2.5. Selective Pressure Detection

The alignments and consensus trees were used to perform the analysis of selective pressure. The analyses of selective pressure compared the nonsynonymous/synonymous substitution ratios (ω = dN/dS) [34] based on the phylogeny with the codon-based maximum likelihood (CodeML) method in the PAML4.7 package [35]. A Values of ω < 1, =1, and >1 correspond to purifying selection, neutral evolution, and positive selection, respectively. The improved branch-site model A (test 2) was performed for every gene in each foreground lineage. Additionally, all the positively selected sites in branch-site models were identified by using Bayes Empirical Bayes (BEB) analysis with posterior probabilities of ≥0.80. Likelihood ratio tests (LRTs) were used to evaluate the significance of differences between the two nested models following a Chi-square distribution by calculating twice the log-likelihood (2ΔL) of the difference. The degrees of freedom refer to the difference in the number of free parameters between models.

## 3. Results

### 3.1. Mitogenome Organization and Base Composition

**Figure 1:**
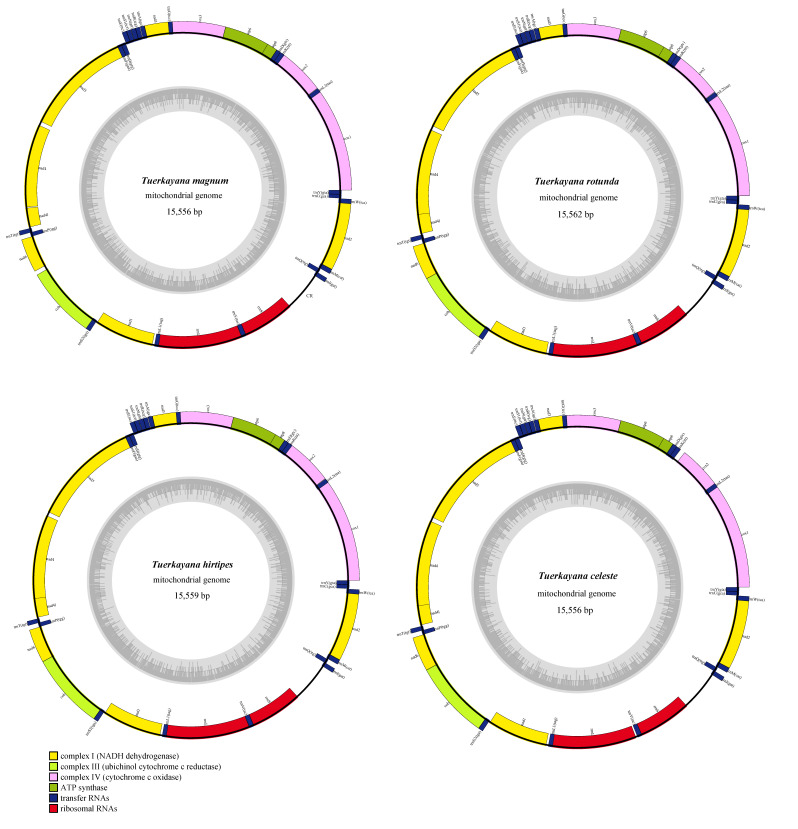
Mitochondrial genome maps of *Tuerkayana*. Protein-coding genes are color-coded (*cox*: lavender; *nad*: yellow; *atp*: green; *cob*: kelly); rRNA genes are in red; tRNA genes are in blue. Abbreviations of protein-coding genes are: *atp6* and *atp8* for ATP synthase subunits 6 and 8, *cox1-3* for *cytochrome oxidase subunits 1-3*, *cob* for *cytochrome b*, *nad1-6* and *nad4l* for *NADH dehydrogenase subunits 1-6* and 4 L, *rrnL* and *rrnS* for large and small rRNA subunits, CR for control region.

The current study sequenced and fully annotated the mitogenome of four species (*T. magnum*, *T. rotundum*, *T. hirtipes*, *T. celeste*) in *Tuerkayana*. The mitogenomes in *Tuerkayana* are 15,556 (*T. magnum*, *T. celeste*), 15,559 (*T. hirtipes*), 15,562 (*T. rotundum*) base pairs (bp) long. Each species has a standard set of 37 genes, including 13 PCGs, 2 rRNAs, 22 tRNAs, and a control region (CR). The distribution was the typical of Decapoda, with 23 genes distributed on the heavy (+) strand, and 14 genes distributed on the light (−) strand (Figure 1 and Table S3). An amount of 14 overlapping regions existed among the mitochondrial genes of species in *Tuerkayana*, and these ranged from 1 bp to 25 bp. The number of overlapping bases is the same except for the difference in *T. magnum* between *cox2* and *trnK*. The longest overlapping region existed between *trnL1* and *rrnL* (Table S3). In addition, a total of 803 (*T. hirtipes*) and −828 (*T. celeste*) bp non-coding regions were present in the mitogenomes of *Tuerkayana*, of which 181 (*T. hirtipes*) −208 (*T. celeste*) bp were distributed in 15 intergenic spacers. The other longest non-coding regions, the CRs, were identically situated between *rrnS* and *trnI* with high A + T content (76.01–79.56%).

The nucleotide bias, which occurred in high A and T representation, was the general characteristic of metazoan mitogenomes [36]. These characteristics resulted in a subsequent bias in homologous encoded amino acids. The AT contents of mitogenome in *Tuerkayana* are high (70.23–71.48%), as is typical of species in Brachyuran (Table S3). In Figure S1, the genera of the Gecarcinidae family are represented separately in a three-dimensional Cartesian coordinate system (*X*-axis represents AT-skew, *Y*-axis represents GC-skew, and *Z*-axis represents AT content). The GC-skews were negative, indicating that Cs were more abundant than Gs. AT-skew and GC-skew resulted from the nucleotides being distributed differentially between the two DNA strands, which caused further DNA strand asymmetry [37].

### 3.2. Protein-Coding Genes and Codon Usage

PCGs (protein-coding genes) in *Tuerkayana* occupied approximately 71.77–71.82% (11,165 bp, 111,72 bp), including one *cytochrome b* (*cob*), two *ATP synthase* (*atp6* and *atp8*), seven *NADH dehydrogenases* (*nad1-6* and *nad4l*), and three *cytochrome c oxidases* (*cox1-3*). The sizes of the 13 PCGs ranged from 159 bp (*atp8*) to 1719 bp (*nad5*) (Table S3). Similar to a typical mitogenome in Brachyura, 9 PCGs were encoded in the heavy strand (*cox1-3*, *cob*, *atp6*, *atp8*, *nad2-3*, *and nad6*), and 4 PCGs were encoded on the light strand (*nad1*, *nad4-5,* and *nad4l*) in every mtDNA of *Tuerkayana*. The start codon of the 13 PCGs used the typical start codon ATN, embracing ATG, ATA, and ATT. In addition, the stop codon of PCGs also used the representative stop codon TNN, including TAA, TAG, and the incomplete stop codon: T (*cox2* in *T. magnum* and *cob*). The peculiar incomplete stop codon (T) was possibly completed as TAA through post-transcriptional polyadenylation and would not affect the normal synthesis of amino acids [38].

**Figure 2:**
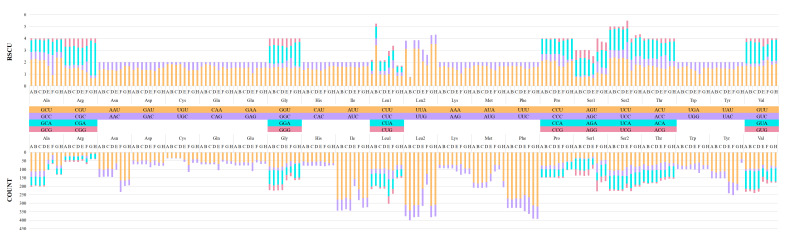
The relative synonymous codon usage (RSCU) in the mitogenomes of Tuerkayana. A: Tuerkayana magnum; B: Tuerkayana rotundum; C: Tuerkayana hirtipes; D: Tuerkayana celeste; E: Cardisoma armatum; F: Cardisoma carnifex; G: Gecarcoidea lalandii; H: Gecarcoidea natalis.

**Figure 3:**
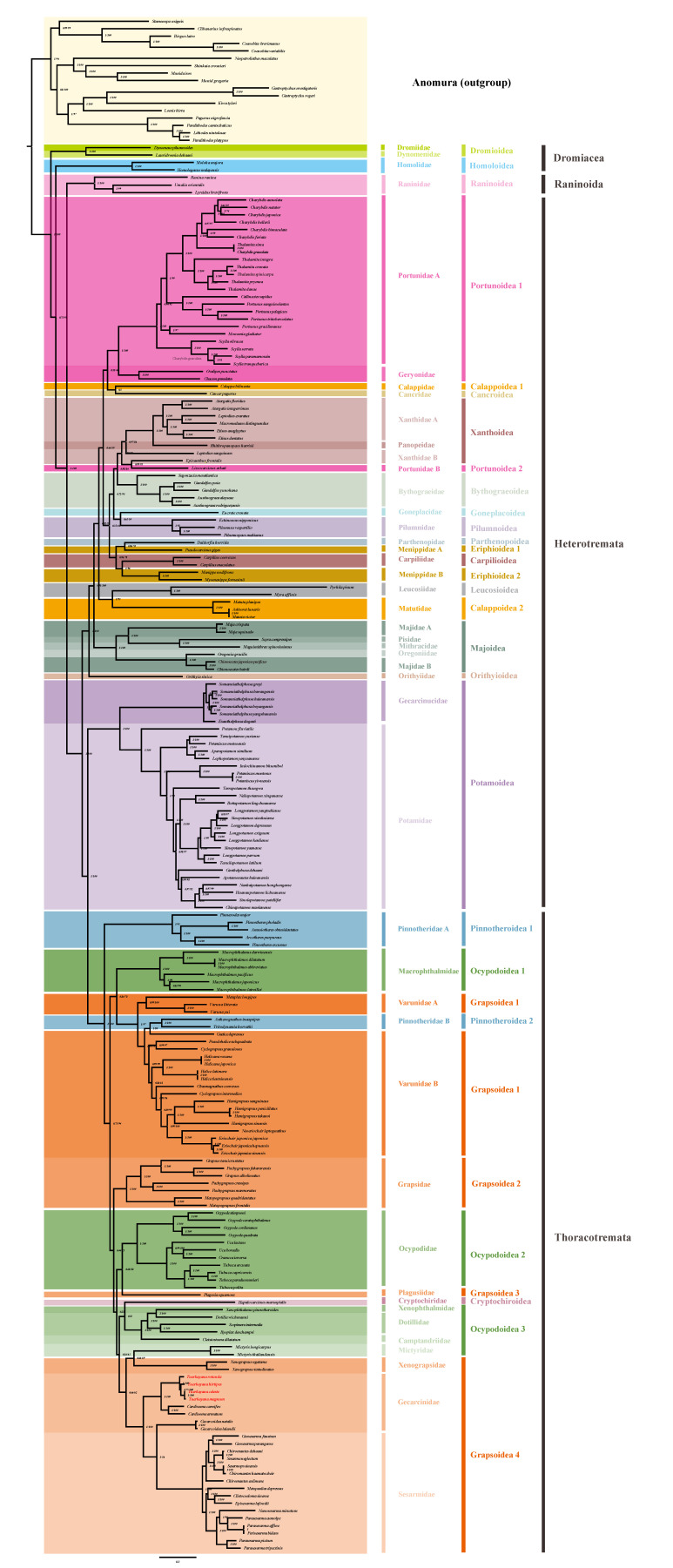
The phylogenetic tree was inferred from the nucleotide sequences of 13 mitogenome PCGs using BI and ML methods, including 189 species of Brachyuran belonging to 39 families and 17 Anomura species as the outgroup. Numbers on branches indicate posterior probabilities (BI) and bootstrap values (ML). The dashed lines on the right represent the families and superfamilies of these species, respectively.

RSCU (Relative Synonymous Codon Usage) is a reference value to evaluate the frequency of codons encoding the same amino acid. When the RSCU results were higher than 1, this suggested that the codon appears many times and with a high frequency. On the contrary, a value of less than one indicates that the frequency of this codon is low and the number of occurrences is small. As shown in Figure 2, the RSCU of single amino acids of Gecarcinidae were compared, and they were all significantly different, especially Leucine (Leu) and Serine (Ser) in terms of the two patterns in the first codons (Leu: CUN, UUA/UUG; Ser: AGN, UCN). The RSCU ratio of each species varies greatly, as UUA, UCU, CCU, and GCU are used relatively frequently, and the frequency of CUG, CCG, GCG, and UGC was low. According to the results, the RSCU values varied widely, indicating that there is a great bias in the usage frequency of codons. The RSCU of NNU and NNA codons were larger than one, indicating that the codons with the third site of the A and T base were used more frequently. This bias in codon usage is consistent with a strong AT bias in the third site of the codon of the protein-coding gene. In addition, we counted the single amino acid usage count. A frequency of 20 amino acids showed a strong bias. The frequencies of leucine (Leu), isoleucine (Ile), phenylalanine (Phe), and serine (Ser) were high. These amino acids were all composed of T or TA. The frequencies of arginine (Arg), aspartate (Asp), and cysteine (Cys) determined by CG-rich base codons were relatively low. This phenomenon is consistent with the mitochondrial genome showing strong AT bias (Figure 3).

### 3.3. Transfer RNAs, Ribosomal RNAs, and CR

The mitogenomes of *Tuerkayana* all included 22 tRNAs, ranging from 62 bp (*trnC* in *T. magnum* and *T. celeste*) to 73 bp (*trnV*) in size. Eight tRNA genes were distributed on the light strand [*trnP*, *trnQ*, *trnV*, *trnC*, *trnY*, *trnH*, *trnL1* (CUN), and *trnF*], and the other 14 tRNAs were distributed on the other strand (Figure 1 and Table S3), these distributions were consistent with other Gecarcinidae crabs. As shown in Figures S2 and S3, the majority of tRNAs exhibited typical cloverleaf structures. However, the *trnS* (AGN) lacked the entire dihydrouridine (DHU) arm, which was simplified as a loop. The absence of the DHU arm in the secondary structure of *trnS* (AGN) is common in the mitogenome of metazoans [39,40]. Previous studies have verified that the lack of the D-arm does not impact the function of tRNA in metazoans. Additionally, many mismatches (G-U, A-C, U-U, C-U) have been found in tRNAs, most of which are G-U pairs. This often occurs in tRNAs of other Crustacea species [41], and these mismatches have been amended in the tRNA modification [42].

Mitogenomes of all species in Gecarcinidae showed that the small coding subunit (*12SrRNA*) and large coding subunit (*16SrRNA*) were separated by *trnV*. In addition, all were located on the light strand, which is also a characteristic shared by most species of Brachyura. Due to the specific function of rRNAs, their sequences are often conserved, such that the secondary structure and three-dimensional composition are not significantly altered (Figure S4).

The CR typically was heavily A + T biased. Its length was 620 (*T. celeste*) −622 (*T. hirtipes*) bp with an A+T content of 76.01% (*T. magnum*) −78.58% (*T. rotundum*). The AT-skew and GC-skew in CR were −0.072–0.074 and −0.306–0.315, respectively. On account of in-depth research undertaken in recent years, CR is no longer considered a pure non-coding. However, its specific function remains to be studied. There were many conserved motifs, such as the poly T-stretch, TA(A)n-like stretch, G(A)nT motif, TATA motif, and hairpin loop structures (Figure S5), and these motifs have been identified as initiation sites for replication and transcription [43]. Notably, Tandem Repeats Finder was used to search for tandem repeats, but none were found, which is unusual in crustaceans.

### 3.4. Phylogenetic Relationships

The paucity of existing mitochondrial genome data constrained the scope of this study. Complete data were only available for the *Tuerkayana* we sequenced. For other species belonging to the same family, information was limited to *cox1* and *16SrRNA* sequences available in the database. In order to facilitate a more comprehensive examination of the taxonomic relationships involving *Tuerkayana*, we reconstructed two single-gene phylogenetic trees (Figure S6 and Table S1) of *cox1* and *16SrRNA* sequences from 11 different species belonging to the Gecarcinidae family (including species from *Discoplax*, *Tuerkayana,* and *Cardisoma*) and a Menippidae species (*Pseudocarcinus gigas*). In the two single gene phylogenetic trees, three genera *Tuerkayana*, *Discoplax,* and *Cardisoma* were closely related and appeared to be monophyletic, forming the relationship of (*Cardisoma* (*Discoplax* + *Tuerkayana*)).

In addition, two types of phylogenetic trees (ML and BI tree) were reconstructed with complete mitogenomes in order to further investigate the phylogenetic position of *Tuerkayana* and Gecarcinidae within Brachyuran. This phylogenetic tree included 189 Brachyuran crabs belonging to 39 families and 17 Anomura species as the outgroup (Table S1). The phylogenetic tree was estimated using the dataset of nucleotide sequences and amino acid sequences of 13 PCGs. In front of the building, the substitution saturation of PCGs was measured by DAMBE (Figure S7), all *iss* were smaller than *iss.c*, indicating that PCGs were not saturated and contained accurate phylogenetic information. Through BI and ML methods, most of the same topological structures were produced from nucleotide sequences and amino acid sequences, respectively. Based on the analysis of support values, the BI topologies were used to present both support values, including the bootstrap values for the ML tree (BS) and the posterior probabilities for Bayesian analysis (BPP), to show the results of nucleotide sequences (N-tree) and amino acid sequences (AA-tree) separately (Figure 2 and Figure S8).

The phylogenetic relationships of *Tuerkayana* can be inferred from its representation in the N-tree as (*T. rotundum* (*T. hirtipes* (*T. celeste* + *T. magnum*))) and in the AA-tree as ((*T. rotundum* + *T. hirtipes*) (*T. celeste* + *T. magnum*)). Both of these topologies support the monophyly of *Tuerkayana* and imply a close evolutionary affinity between *T. celeste* and *T. magnum*.

Notably, the N-tree and AA-tree display divergent phylogenetic information. The first and fundamental question is “Is the G family monophyletic?”. The answer to this question depends mainly on the position of the genus *Gecarcoidea* in the phylogenetic trees (Figure S6). According to the N-tree, the *Gecarcoidea* and Sesarmidae exhibit a sister group relationship with high BPP and low BS, indicating that Gecarcinidae is partially paraphyletic with respect to Sesarmidae, presenting a ((*Tuerkayana* + *Cardisoma*) (*Gecarcoidea* + Sesarmidae)) topology. Thus, Gecarcinidae appears to be polyphyletic. However, the AA-tree presents a different scenario, as it shows that Gecarcinidae is monophyletic, featuring a (*Gecarcoidea* (*Tuerkayana* + *Cardisoma*)) topology with robust support values for both BS and BPP. To further investigate this issue, a genetic distance analysis (p-distance) was conducted (Figure S9 and Table S4). The results indicate that the p-distance between *Gecarcoidea* and (*Tuerkayana* + *Cardisoma*) is 0.1485–0.1659 (average 0.1587), whereas the p-distance between *Gecarcoidea* and Sesarmidae is 0.1512–0.1711 (average 0.1602). The p-distance between Gecarcinidae and Sesarmidae was 0.1773. Based solely on genetic distance values, the p-distance between *Gecarcoidea* and (*Tuerkayana* + *Cardisoma*) was slightly smaller than between Gecarcinidae and Sesarmidae, indicating a closer relationship between *Gecarcoidea* and the genera *Tuerkayana* and *Cardisoma*, which supports the current classification status of Gecarcinidae. Nevertheless, further studies combining morphology and biogeography are necessary to validate this conclusion. Additionally, a notable divergence can be observed in the classification of Gecarcinidae within the N-tree and AA-tree (Figure S6). In the N-tree, Gecarcinidae is closely associated with Sesarmidae and Xenograpsidae, which are typically positioned in the Grapsoidea 4. In different forms, the AA-tree exhibits a more intricate configuration, with Gecarcinidae classified as (Gecarcinidae (Camptandriidae (Dotillidae (Dotillidae + Xenophthalmidae)) + Sesarmidae)) rather than associated solely with Sesarmidae. The utilization of the genetic distance analysis reveals that the distance between Gecarcinidae or Sesarmidae and Ocypodoidea 4 is greater than between Gecarcinidae and Sesarmidae, providing evidence to support the closer relationship between Gecarcinidae and Sesarmidae and thus justifying the N-tree topology. Additionally, comparisons made with previous studies lent further credence to this result [44,45]. Third, despite the lack of substantial differences in the overall status of the superfamily, the distribution of individual families within it has resulted in significant variations. Following a thorough comparison with the phylogenetic tree presented in previous studies, we posit that the N-tree represents a more precise depiction of the evolutionary relationships between families. Consequently, we elected to concentrate on the N-tree.

Having been added to the dataset, 33 of the 39 families included in this tree formed a monophyletic clade, and from the other families arose branches. This finding is not consistent with previous research [46,47]. Therefore, it is necessary to increase the mitogenome of species for the research of phylogeny. In addition, *Lissocarcinus arkati* (Portunidae species) occurred unexpectedly in Xanthidae. Through the analysis and comparison of 13 PCGs, *L. arkati* was determined as closer to *Eriphia* species, and we speculated that *L. arkati* should belong to *Eriphia* or that there may have been an identification error or a contamination in the original study. Furthermore, at the superfamily level, Ocypodoidea and Grapsoidea were polyphyletic, with Grapsoidea and Ocypodoidea mutually intersecting. Many studies have suggested that Grapsoidea and Ocypodoidea could be polyphyletic and combined into a large taxon [48,49,50]. This conjecture is consistent with our phylogenetic analysis, and the reconstruction of the phylogenetic tree in this study used more location data than in previous studies, enhancing the persuasiveness of this conjecture. At the same time, some taxonomists have proposed that the morphological classification of Ocypodoidea and Grapsoidea is indeed inconsistent with molecular phylogeny, but the creation of these superfamilies was primarily for practical purposes and convenience rather than phylogenetic accuracy [51,52].

### 3.5. Mitogenome Gene Rearrangement

The mitogenome order of species in Gecarcinidae is presented in Figure S10. This figure shows that all the species in the Gecarcinidae have the same gene order pattern as the ancestors of Brachyura. This is consistent with a previous study regarding the evolution of Brachyura (Crustacea: Decapoda) based on mitochondrial arrangement and gene order rearrangements [4].

**Figure 4:**
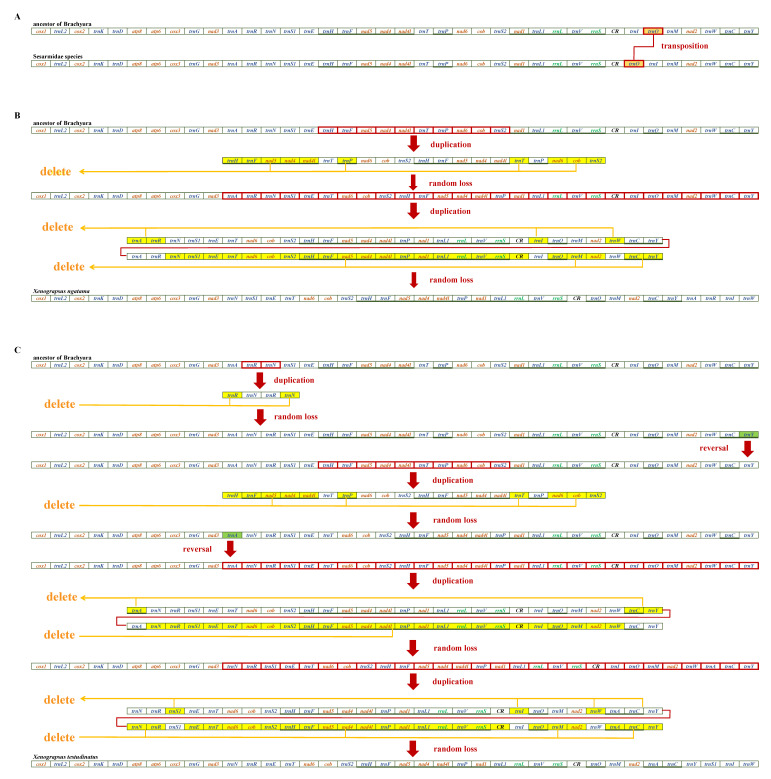
(**A**) Evolutionary procession from the ancestors of Brachyura to Sesarmidae; (**B**) Evolutionary procession from the ancestors of Brachyura to *Xenograpsus ngatama*. (**C**) Evolutionary procession from the ancestors of Brachyura to *Xenograpsus testudinatus*.

The mitogenome arrangements of a Grapsoidea lineage (Sesarmidae and Xenograpsidae) have been researched (Figure S10) and were determined to be located in the same branch as “Grapsoidea 4” (Figure S6). We studied the sequence evolution of genes from three aspects (Figure 4), namely the (A) evolutionary process from the ancestor of Brachyura to Sesarmidae, (B) evolutionary process from the ancestor of Brachyura to *Xenograpsus ngatama*, and (C) evolutionary process from the ancestor of Brachyura to *Xenograpsus testudinatus*. The mitogenome gene arrangements of all Gecarcinidae species were the same. It is clear, therefore, that the mitogenome arrangements of Sesarmidae species are identical and differ from the ancestral arrangement of the Brachyura only by the inversion of *trnI*-*trnQ*, which has been described in previous studies of Sesarmidae species [53,54].

As a semi-terrestrial family of Grapsoidea, the species of Xenograpsidae usually inhabit the harsh environment of the Epipelagic thermal spring ecosystem [55]. Xenograpsidae contained one genus (*Xenograpsus*), and three known species are included in this category, two (*X. ngatama* and *X. testudinatus*) of which have published complete mitogenomes [56]. In this study, CREx was used to preliminarily predict the putative evolutionary rearrangement mechanisms in *X. ngatama* and *X. testudinatus*. The results of this analysis are summarized in Figure 4B. Two tandem duplication-random loss (TDRL) events were inferred from the Brachyura ancestor to *X. ngatama*. The first TDRL event occurred between *trnH* and *trnS2.* Then, *trnH-trnF-nad5-nad4-nad4l*, *trnP*, *trnT*, and *nad6-cob-trnS2* were randomly absent. The second TDRL event went from *trnA* to *trnY*. Then, six redundant fragments were deleted (*trnA-trnR*, *trnI*, *trnW*, *trnN-trnS1-trnE-trnT-nad6-cob-trnS2-trnH-trnF-nad5-nad4-nad4l-trnP-nad1-trnL1-rrnL-trnV-rrnS-CR*, *trnQ-trnM-nad2*, *trnC-trnY*). Moreover, from Brachyura ancestors to *X. testudinatus*, a complex genetic rearrangement was determined, which is illustrated in Figure 4C. This genetic rearrangement is more complicated than that of the Brachyura ancestor to *X. ngatama*, which started with a small-scale TDRL with *trnN* and *trnR* transposed and accompanied by *trnY* reversed. Subsequently, the TDRL event went from *trnH* to *trnS2*. Then four redundant fragments were lost (*trnH-trnF-nad5-nad4-nad4l*, *trnP*, *trnT*, *nad6*-*cob-trnS2*), with *trnA* reversed. After two consecutive TDRLs and gene reversals, two large-range TDRLs were experienced again, which changed the position of *trnA*, *trnC*, *trnY* and *trnS1*, *trnI*, *trnW*, respectively. The three families are closely related but have completely different rearrangement mechanisms.

### 3.6. Selective Pressure in Gecarcinidae

To explore the adaptive evolution of Brachyura in the mitochondrial genes and assess the evolutionary patterns of PCGs, the branch-site model was used to detect selective pressure in individual codons for 206 crabs (Table S1) with CodeML, and the dN/dS(ω) were calculated. The codon *nad6* was found under positive selection, where LRTs of the branch-site model A were statistically significant (Table 1). Except for *nad6*, the ω values of the other 12 PCGs were far lower than one, indicating that the other 12 PCGs had a low evolutionary rate, and *nad6* was subjected to strong positive selection (*p* << 0.05, ω2 >> 1).

## 4. Discussion

In this study, the complete mitogenome of four species in *Tuerkayana* was determined and described. Based on the phylogenetic tree constructed from the most complete dataset of mitogenomes in Brachyura to date, the classification within Brachyura was further investigated, stabilizing the phylogenetic positions of *Tuerkayana* and Gecarcinidae and furthered our understanding. Our four primary conclusions are given below.

First, *Tuerkayana* is an independent genus. Based on morphological analysis, four species previously assigned to *Discoplax* (*T. celeste* and *T. magnum*) and *Cardisoma* (*T. rotundum* and *T. hirtipes*) were divided into the new genus, *Tuerkayana*. The specific manifestation is proepistome dome-shaped wide but low, pleonal somite six broad and short, telson short, bluntly tipped, no suborbital ridge, and no stridulatory apparatus [5]. Based on the results of the two single gene phylogenetic trees, it is evident that the three genera (*Tuerkayana*, *Discoplax,* and *Cardisoma*) are closely related and show monophyly of each branch. This consequence is consistent with the results of morphological studies, which support *Tuerkayana* as an independent genus.

Secondly, the mitogenomes in Gecarcinidae are structurally stable, indicating a common origin or common derivation from adaptation to a similar terrestrial environment. In Gecarcinidae, the mitogenomes have 37 genes. Their size varied from 15,545 (*G. natalis*) to 15,597 bp (*C. carnifex*), with a maximum difference of only 52bp [5,57,58]. These lengths are smaller in metazoans, and the genes are compact. The range of A + T content of species in Gecarcinidae is from 68.86% to 75.22%, with an abundance of A and T. As a highly mutated region in the mitogenome, CR is the most prone to gene mutation and length change. While in Gecarcinidae, the CR region is highly conserved, and the size, position (*rrnS*-CR-*trnI*), and stem–loop structures of CR did not demonstrate significant variation. Research of the complete mitogenome demonstrates that the gene order in vertebrates is almost fixed, and gene rearrangement rarely occurs [59]. In contrast, in invertebrates, the probabilities of gene rearrangement is significantly higher, and a variety of gene rearrangement models are derived [60]. However, the genetic order of Gecarcinidae is the same as that of the presumed ancestor of Brachyura. As the most derived family within Brachyura, it exhibited no genetic rearrangement, which is relatively rare among invertebrates. All of the above evidence suggests that the mitogenome of Gecarcinidae is structurally stable. We conjecture that this is on account of common origin or derived from adaptations to a similar terrestrial environment.

Third, *Gecarcoidea* belongs to the monophyletic Gecarcinidae. However, the monophyly of Gecarcinidae remains uncertain due to the inconsistent positions of *Gecarcoidea* on the N-tree and AA-tree. Although a preliminary analysis based on genetic distance indicates a closer relationship between *Gecarcoidea* and other species in Gecarcinidae, this inference lacks conclusive evidence. To resolve the taxonomic position of Gecarcinidae, additional evidence from morphology and geographic distribution must be considered. Morphologically, species of Gecarcinidae can be distinguished from other Grapsoidea by the unique characteristics of zoea larvae, antennal and telson morphology, and setation of the second maxilliped endopod [51]. Geographically, members of Gecarcinidae are restricted to tropical island regions (such as Indonesia, the Spratly Islands, the Philippines) and typically inhabit damp crevices, migrating to the sea only during the breeding season. In contrast, Sesarmidae have a much broader distribution, encompassing nearly all coastal regions in tropical and subtropical areas. Given these factors, we tentatively support the monophyly of Gecarcinidae while recognizing that the evidence remains inconclusive. Furthermore, the presence of polyphyly in the N-tree raises the possibility of homology and a close relationship between Gecarcinidae and Sesarmidae.

Fourth, *nad6* may play a significant role in the adaptation within Gecarcinidae. According to its terrestrial characteristics, Gecarcinidae would inevitably face selection pressure to adapt to the habitat, so the branch-site has been conducted here. It is remarkable that *nad6* was subjected to strong positive selection (*p* << 0.05, ω2 >> 1); *nad6* is an essential part of the NADH dehydrogenase (complex I) whose alteration can have a significant impact on organisms [61]. NADH dehydrogenase is an important enzyme that catalyzes the oxidation of NADH into NAD^+^. NAD^+^ maintains redox homeostasis, energy metabolism, DNA repair, gene expression, adaptive stress responses, metabolism, mitochondrial homeostasis, and cellular bioenergetics [62,63,64]. The dynamic NAD^+^ rewires biological processes with post-synthesis modification of fundamental biomolecules to enable cells the adaption to environmental changes [65]. Given that Gecarcinidae repeatedly transitioned from marine to terrestrial environments and suffered from hypoxia stress, the larger numbers of nonsynonymous substitutions, which accumulated in *nad6*, indicate its critical effect on the adaptive process. The adaptive evolution of *nad6* has been previously reported in the study of mammals (*Equus caballus*) [66] subjected to a low temperature and hypoxia environment. These results indicate that *nad6* was significantly determined by the regulation of hypoxia. In consequence, unusual selection pressures acting at the molecular level for organisms that were subjected to hypoxia stress would be disclosed by comparative analyses of complete mitogenomes in Gecarcinidae. This study lays an important foundation for exploring the process of terrestrial Gecarcinidae and provides new insight into possible molecular adaptation mechanisms in crabs under hypoxia. However, the analyses of molecular evolution that this study provided could be strengthened. Only 1 out of 13 genes was found to indicate a signal of selection that could be random or a species-specific pattern. Future analyses of the biochemical or protein structure are, therefore, needed.

## 5. Conclusions

The complete mitogenome sequences of four species in *Tuerkayana* were determined and analyzed for the first time. The most comprehensive phylogenetic tree of Brachyura involving 206 crabs was constructed. The research evaluated the phylogenetic position of *Tuerkayana* and Gecarcinidae in Brachyuran, further supported the establishment of *Tuerkayana* and the division of the genus in Gecarcinidae at the mitochondrial level, and investigated the monophyly of Gecarcinidae. The mitochondrial genome structure of Gecarcinidae is stable. The strong selective pressure shown in *nad6* suggested that it may play a crucial part in the evolution of Gecarcinidae.

## Figures and Tables

**Table 1:** Selective pressure analyses (branch-site model) of mitochondrial protein-encoding genes (13 genes) in all crabs datasets.

Gene	Model	lnL	2lnL	*p* Level	Parameters	Positive Selected Sites (Posterior Probabilities)
*atp8*	Ge					
	ma	−14,486.44134			ω0 = 0.087 ω1 = 1.0 ω2 = 1.0	19 I 0.852;
	ma0	−14,486.44134	0	1	ω0 = 0.087 ω1 = 1.0 ω2 = 1.0	
*cox2*	Ge					
	ma	−39,595.89288			ω0 = 0.018 ω1 = 1.0 ω2 = 1.628	
	ma0	−39,595.89284	−7.4 × 10^−5^	1	ω0 = 0.018 ω1 = 1.0 ω2 = 1.0	
*cox3*	Ge					
	ma	−44,309.9257			ω0 = 0.016 ω1 = 1.0 ω2 = 1.605	
	ma0	−44,309.92564	−0.00012	1	ω0 = 0.016 ω1 = 1.0 ω2 = 1.0	
*cob*	Ge					
	ma	−69,109.49107			ω0 = 0.017 ω1 = 1.0 ω2 = 1.0	
	ma0	−69,109.49113	0.00012	0.991259787	ω0 = 0.017 ω1 = 1.0 ω2 = 1.0	
*cox1*	Ge					
	ma	−77,798.40391			ω0 = 0.008 ω1 = 1.0 ω2 = 2.421	
	ma0	−77,798.40376	−0.000292	1	ω0 = 0.008 ω1 = 1.0 ω2 = 1.0	
*atp6*	Ge					
	ma	−42,775.44489			ω0 = 0.02 ω1 = 1.0 ω2 = 11.004	
	ma0	−42,775.44974	0.009696	0.921560467	ω0 = 0.02 ω1 = 1.0 ω2 = 1.0	
*nad4l*	Ge					
	ma	−19,516.44828			ω0 = 0.023 ω1 = 1.0 ω2 = 1.0	
	ma0	−19,514.55719	−3.782182	1	ω0 = 0.023 ω1 = 1.0 ω2 = 1.0	
*nad1*	Ge					
	ma	−55,412.74594			ω0 = 0.017 ω1 = 1.0 ω2 = 1.0	
	ma0	−55,412.74594	0	1	ω0 = 0.017 ω1 = 1.0 ω2 = 1.0	
*nad3*	Ge					
	ma	−24,126.5674			ω0 = 0.026 ω1 = 1.0 ω2 = 1.0	
	ma0	−24,126.5674	2 × 10^−6^	0.998871621	ω0 = 0.026 ω1 = 1.0 ω2 = 1.0	
*nad2*	Ge					
	ma	−90,455.6106			ω0 = 0.042 ω1 = 1.0 ω2 = 1.0	
	ma0	−90,455.6106	0	1	ω0 = 0.042 ω1 = 1.0 ω2 = 1.0	
*nad5*	Ge					
	ma	−123,998.0812			ω0 = 0.034 ω1 = 1.0 ω2 = 1.0	
	ma0	−123,998.0812	0	1	ω0 = 0.034 ω1 = 1.0 ω2 = 1.0	
*nad4*	Ge					
	ma	−91,823.48875			ω0 = 0.03 ω1 = 1.0 ω2 = 1.0	386 I 0.728;
	ma0	−91,823.48875	0	1	ω0 = 0.03 ω1 = 1.0 ω2 = 1.0	
*nad6*	Ge					
	ma	−45,717.39912			ω0 = 0.043 ω1 = 1.0 ω2 = 137.937	28 L 0.686; 36 V 0.556; 88 I 0.997;
	ma0	−45,721.31165	7.825062	0.005152668	ω0 = 0.043 ω1 = 1.0 ω2 = 1.0	

## Data Availability

The data that support this study are available at NCBI and are provided in Table S1.

## Supplementary Materials

The following supporting information can be downloaded at: https://www.mdpi.com/article/10.3390/biology12070974/s1, Figure S1: The three-dimensional Cartesian coordinate system within Gecarcinidae. X-axis represents AT-skew, Y-axis represents GC-skew, and Z-axis represents AT content. *Tuerkayana* are represented by circles, *Cardisoma* by triangles, and *Gecarcoidea* by squares; Figure S2: Predicted secondary cloverleaf structure for the tRNAs of *Tuerkayana magnum*; Figure S3: Predicted secondary cloverleaf structure for the tRNAs of *Tuerkayana rotunda*, *Tuerkayana hirtipes*, and *Tuerkayana celeste*; Figure S4: Predicted secondary structure for *16SrRNA* and *12SrRNA* in *Tuerkayana*; Figure S5: Predicted secondary structure for CR (control region) in *Tuerkayana*; Figure S6: Nucleotide substitution saturation analysis of PCGs. X-axis represents F84 distance and Y-axis represents s and v; Figure S7: (A,B): Two single gene phylogenetic trees were inferred from *cox1* and *16SrRNA* using BI and ML methods, including 11 Gecarcinidae species (including all *Discoplax* species, *Tuerkayana* species and *Cardisoma* species have been sequenced) and a Menippidae species (*Pseudocarcinus gigas*). Numbers on branches indicate posterior probability (BI) and bootstrap values (ML). (C,D): The configurational difference of trees in nucleotide and amino acid sequences by PCGs, especially the taxonomic status of Gecarcinidae; Figure S8: The phylogenetic tree was inferred from the amino acid sequences of 13 mitogenome PCGs using BI and ML methods; Figure S9: The genetic distance analysis (p-distance) between species in Gecarcinidae and Sesarmidae; Figure S10: The mitogenome genetic sequence of species in Gecarcinidae, Sesarmidae, Xenograpsidae, and the ancestor of Brachyura. All genes are transcribed from left to right. CR represents control region. *rrnL* and *rrnS* are the large and small ribosomal RNA subunits, respectively; Table S1: The GenBank accession numbers of 189 Brachyura species and 17 Anomura species in this study; Table S2: Sequences of universal primers; Table S3: Summary of mitochondrial genomes of *Tuerkayana magnum*, *Tuerkayana rotunda*, *Tuerkayana hirtipe*, *Tuerkayana celeste*; Table S4: Estimates of Evolutionary Divergence.
