# Supplementary material for: Molecular Phylogeny and Evolution of the Tuerkayana (Decapoda: Brachyura: Gecarcinidae) Genus Based on Whole Mitochondrial Genome Sequences
Source: Biology (Basel). 2023 Jul 8;12(7):974. doi: 10.3390/biology12070974 (PMC10376310; doi:10.3390/biology12070974)
Supplement: Supplementary file 1 [file biology-12-00974-s001.zip › Figure S10.pdf]

ancestor of Brachyura

cox1 | trnL2 | cox2 | trnK | trnD | atp8 | atp6 | cox3 | trnG | nad3 | trnA | trnR | trnN | trnS1 | trnE | trnH | trnF | nad5 | nad4 | nad4l | trnT | trnP | nad6 | cob | trnS2 | nad1 | trnL1 | rrnL | trnV | rrnS | CR | trnI | trnQ | trnM | nad2 | trnW | trnC | trnY

Gecarcinidae

Tuerkayana magnum

cox1 | trnL2 | cox2 | trnK | trnD | atp8 | atp6 | cox3 | trnG | nad3 | trnA | trnR | trnN | trnS1 | trnE | trnH | trnF | nad5 | nad4 | nad4l | trnT | trnP | nad6 | cob | trnS2 | nad1 | trnL1 | rrnL | trnV | rrnS | CR | trnI | trnQ | trnM | nad2 | trnW | trnC | trnY

Tuerkayana rotunda

cox1 | trnL2 | cox2 | trnK | trnD | atp8 | atp6 | cox3 | trnG | nad3 | trnA | trnR | trnN | trnS1 | trnE | trnH | trnF | nad5 | nad4 | nad4l | trnT | trnP | nad6 | cob | trnS2 | nad1 | trnL1 | rrnL | trnV | rrnS | CR | trnI | trnQ | trnM | nad2 | trnW | trnC | trnY

Tuerkayana hirtipes

cox1 | trnL2 | cox2 | trnK | trnD | atp8 | atp6 | cox3 | trnG | nad3 | trnA | trnR | trnN | trnS1 | trnE | trnH | trnF | nad5 | nad4 | nad4l | trnT | trnP | nad6 | cob | trnS2 | nad1 | trnL1 | rrnL | trnV | rrnS | CR | trnI | trnQ | trnM | nad2 | trnW | trnC | trnY

Tuerkayana celeste

cox1 | trnL2 | cox2 | trnK | trnD | atp8 | atp6 | cox3 | trnG | nad3 | trnA | trnR | trnN | trnS1 | trnE | trnH | trnF | nad5 | nad4 | nad4l | trnT | trnP | nad6 | cob | trnS2 | nad1 | trnL1 | rrnL | trnV | rrnS | CR | trnI | trnQ | trnM | nad2 | trnW | trnC | trnY

Cardisoma carnifex

cox1 | trnL2 | cox2 | trnK | trnD | atp8 | atp6 | cox3 | trnG | nad3 | trnA | trnR | trnN | trnS1 | trnE | trnH | trnF | nad5 | nad4 | nad4l | trnT | trnP | nad6 | cob | trnS2 | nad1 | trnL1 | rrnL | trnV | rrnS | CR | trnI | trnQ | trnM | nad2 | trnW | trnC | trnY

Cardisoma armatum

cox1 | trnL2 | cox2 | trnK | trnD | atp8 | atp6 | cox3 | trnG | nad3 | trnA | trnR | trnN | trnS1 | trnE | trnH | trnF | nad5 | nad4 | nad4l | trnT | trnP | nad6 | cob | trnS2 | nad1 | trnL1 | rrnL | trnV | rrnS | CR | trnI | trnQ | trnM | nad2 | trnW | trnC | trnY

Gecarcoidea lalandii

cox1 | trnL2 | cox2 | trnK | trnD | atp8 | atp6 | cox3 | trnG | nad3 | trnA | trnR | trnN | trnS1 | trnE | trnH | trnF | nad5 | nad4 | nad4l | trnT | trnP | nad6 | cob | trnS2 | nad1 | trnL1 | rrnL | trnV | rrnS | CR | trnI | trnQ | trnM | nad2 | trnW | trnC | trnY

Gecarcoidea natalis

cox1 | trnL2 | cox2 | trnK | trnD | atp8 | atp6 | cox3 | trnG | nad3 | trnA | trnR | trnN | trnS1 | trnE | trnH | trnF | nad5 | nad4 | nad4l | trnT | trnP | nad6 | cob | trnS2 | nad1 | trnL1 | rrnL | trnV | rrnS | CR | trnI | trnQ | trnM | nad2 | trnW | trnC | trnY

Sesarmidae

Clistocoeloma sinense

cox1 | trnL2 | cox2 | trnK | trnD | atp8 | atp6 | cox3 | trnG | nad3 | trnA | trnR | trnN | trnS1 | trnE | trnH | trnF | nad5 | nad4 | nad4l | trnT | trnP | nad6 | cob | trnS2 | nad1 | trnL1 | rrnL | trnV | rrnS | CR | trnQ | trnI | trnM | nad2 | trnW | trnC | trnY

Metopaulias depressus

cox1 | trnL2 | cox2 | trnK | trnD | atp8 | atp6 | cox3 | trnG | nad3 | trnA | trnR | trnN | trnS1 | trnE | trnH | trnF | nad5 | nad4 | nad4l | trnT | trnP | nad6 | cob | trnS2 | nad1 | trnL1 | rrnL | trnV | rrnS | CR | trnQ | trnI | trnM | nad2 | trnW | trnC | trnY

Nanosesarma minutum

cox1 | trnL2 | cox2 | trnK | trnD | atp8 | atp6 | cox3 | trnG | nad3 | trnA | trnR | trnN | trnS1 | trnE | trnH | trnF | nad5 | nad4 | nad4l | trnT | trnP | nad6 | cob | trnS2 | nad1 | trnL1 | rrnL | trnV | rrnS | CR | trnQ | trnI | trnM | nad2 | trnW | trnC | trnY

Perisesarma bidens

cox1 | trnL2 | cox2 | trnK | trnD | atp8 | atp6 | cox3 | trnG | nad3 | trnA | trnR | trnN | trnS1 | trnE | trnH | trnF | nad5 | nad4 | nad4l | trnT | trnP | nad6 | cob | trnS2 | nad1 | trnL1 | rrnL | trnV | rrnS | CR | trnQ | trnI | trnM | nad2 | trnW | trnC | trnY

Parasesarma affine

cox1 | trnL2 | cox2 | trnK | trnD | atp8 | atp6 | cox3 | trnG | nad3 | trnA | trnR | trnN | trnS1 | trnE | trnH | trnF | nad5 | nad4 | nad4l | trnT | trnP | nad6 | cob | trnS2 | nad1 | trnL1 | rrnL | trnV | rrnS | CR | trnQ | trnI | trnM | nad2 | trnW | trnC | trnY

Parasesarma tripectinis

cox1 | trnL2 | cox2 | trnK | trnD | atp8 | atp6 | cox3 | trnG | nad3 | trnA | trnR | trnN | trnS1 | trnE | trnH | trnF | nad5 | nad4 | nad4l | trnT | trnP | nad6 | cob | trnS2 | nad1 | trnL1 | rrnL | trnV | rrnS | CR | trnQ | trnI | trnM | nad2 | trnW | trnC | trnY

Parasesarma pictum

cox1 | trnL2 | cox2 | trnK | trnD | atp8 | atp6 | cox3 | trnG | nad3 | trnA | trnR | trnN | trnS1 | trnE | trnH | trnF | nad5 | nad4 | nad4l | trnT | trnP | nad6 | cob | trnS2 | nad1 | trnL1 | rrnL | trnV | rrnS | CR | trnQ | trnI | trnM | nad2 | trnW | trnC | trnY

Chiromantes haematocheir

cox1 | trnL2 | cox2 | trnK | trnD | atp8 | atp6 | cox3 | trnG | nad3 | trnA | trnR | trnN | trnS1 | trnE | trnH | trnF | nad5 | nad4 | nad4l | trnT | trnP | nad6 | cob | trnS2 | nad1 | trnL1 | rrnL | trnV | rrnS | CR | trnQ | trnI | trnM | nad2 | trnW | trnC | trnY

Sesarmops sinensis

cox1 | trnL2 | cox2 | trnK | trnD | atp8 | atp6 | cox3 | trnG | nad3 | trnA | trnR | trnN | trnS1 | trnE | trnH | trnF | nad5 | nad4 | nad4l | trnT | trnP | nad6 | cob | trnS2 | nad1 | trnL1 | rrnL | trnV | rrnS | CR | trnQ | trnI | trnM | nad2 | trnW | trnC | trnY

Chiromantes dehaani

cox1 | trnL2 | cox2 | trnK | trnD | atp8 | atp6 | cox3 | trnG | nad3 | trnA | trnR | trnN | trnS1 | trnE | trnH | trnF | nad5 | nad4 | nad4l | trnT | trnP | nad6 | cob | trnS2 | nad1 | trnL1 | rrnL | trnV | rrnS | CR | trnQ | trnI | trnM | nad2 | trnW | trnC | trnY

Sesarma neglectum

cox1 | trnL2 | cox2 | trnK | trnD | atp8 | atp6 | cox3 | trnG | nad3 | trnA | trnR | trnN | trnS1 | trnE | trnH | trnF | nad5 | nad4 | nad4l | trnT | trnP | nad6 | cob | trnS2 | nad1 | trnL1 | rrnL | trnV | rrnS | CR | trnQ | trnI | trnM | nad2 | trnW | trnC | trnY

Chiromantes eulimene

cox1 | trnL2 | cox2 | trnK | trnD | atp8 | atp6 | cox3 | trnG | nad3 | trnA | trnR | trnN | trnS1 | trnE | trnH | trnF | nad5 | nad4 | nad4l | trnT | trnP | nad6 | cob | trnS2 | nad1 | trnL1 | rrnL | trnV | rrnS | CR | trnQ | trnI | trnM | nad2 | trnW | trnC | trnY

Xenograpsidae

Xenograpsus ngatama

cox1 | trnL2 | cox2 | trnK | trnD | atp8 | atp6 | cox3 | trnG | nad3 | trnN | trnS1 | trnE | trnT | nad6 | cob | trnS2 | trnH | trnF | nad5 | nad4 | nad4l | trnP | nad1 | trnL1 | rrnL | trnV | rrnS | CR | trnQ | trnM | nad2 | trnC | trnY | trnA | trnR | trnI | trnW

Xenograpsus testudinatus

cox1 | trnL2 | cox2 | trnK | trnD | atp8 | atp6 | cox3 | trnG | nad3 | trnN | trnR | trnE | trnT | nad6 | cob | trnS2 | trnH | trnF | nad5 | nad4 | nad4l | trnP | nad1 | trnL1 | rrnL | trnV | rrnS | CR | trnQ | trnM | nad2 | trnA | trnC | trnY | trnS1 | trnI | trnW
