# Supplementary material for: Molecular Phylogeny and Evolution of the Tuerkayana (Decapoda: Brachyura: Gecarcinidae) Genus Based on Whole Mitochondrial Genome Sequences
Source: Biology (Basel). 2023 Jul 8;12(7):974. doi: 10.3390/biology12070974 (PMC10376310; doi:10.3390/biology12070974)
Supplement: Supplementary file 1 [file biology-12-00974-s001.zip › Figure S4.pdf]

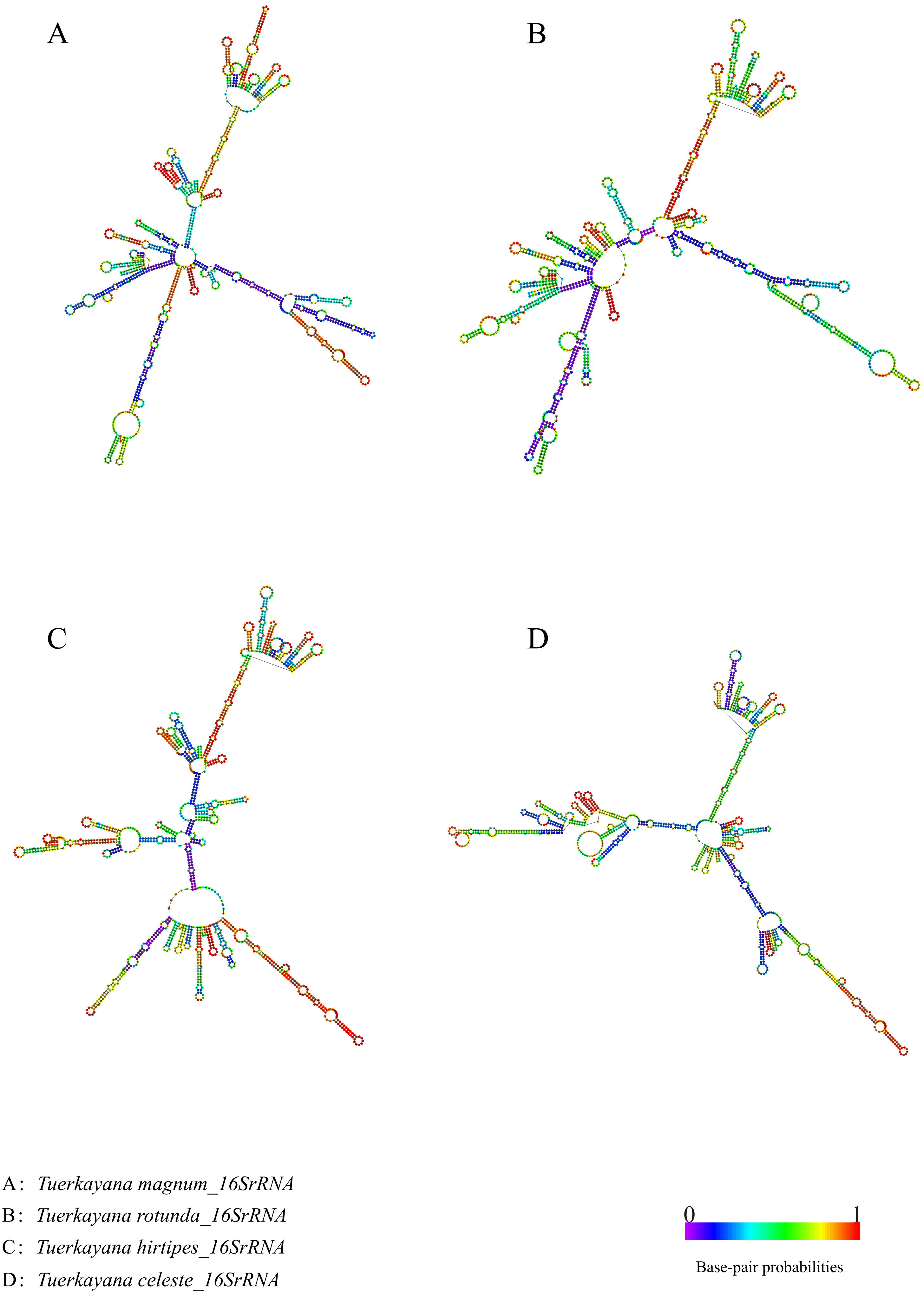

A: *Tuerkayana magnum_16SrRNA*
B: *Tuerkayana rotunda_16SrRNA*
C: *Tuerkayana hirtipes_16SrRNA*
D: *Tuerkayana celeste_16SrRNA*

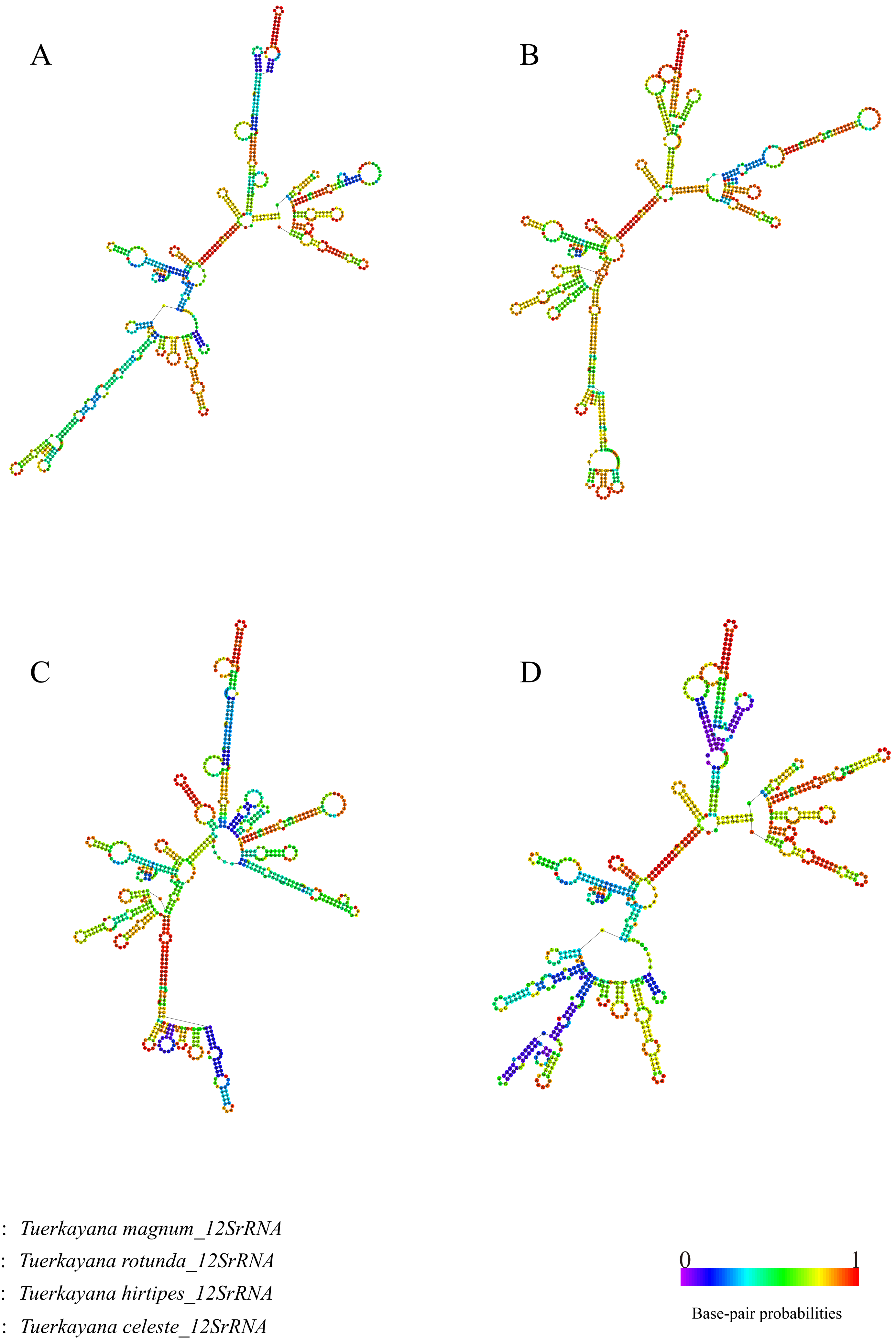

A: *Tuerkayana magnum_12SrRNA*
B: *Tuerkayana rotunda_12SrRNA*
C: *Tuerkayana hirtipes_12SrRNA*
D: *Tuerkayana celeste_12SrRNA*
