# Supplementary material for: Molecular Phylogeny and Evolution of the Tuerkayana (Decapoda: Brachyura: Gecarcinidae) Genus Based on Whole Mitochondrial Genome Sequences
Source: Biology (Basel). 2023 Jul 8;12(7):974. doi: 10.3390/biology12070974 (PMC10376310; doi:10.3390/biology12070974)
Supplement: Supplementary file 1 [file biology-12-00974-s001.zip › Figure S5.pdf]

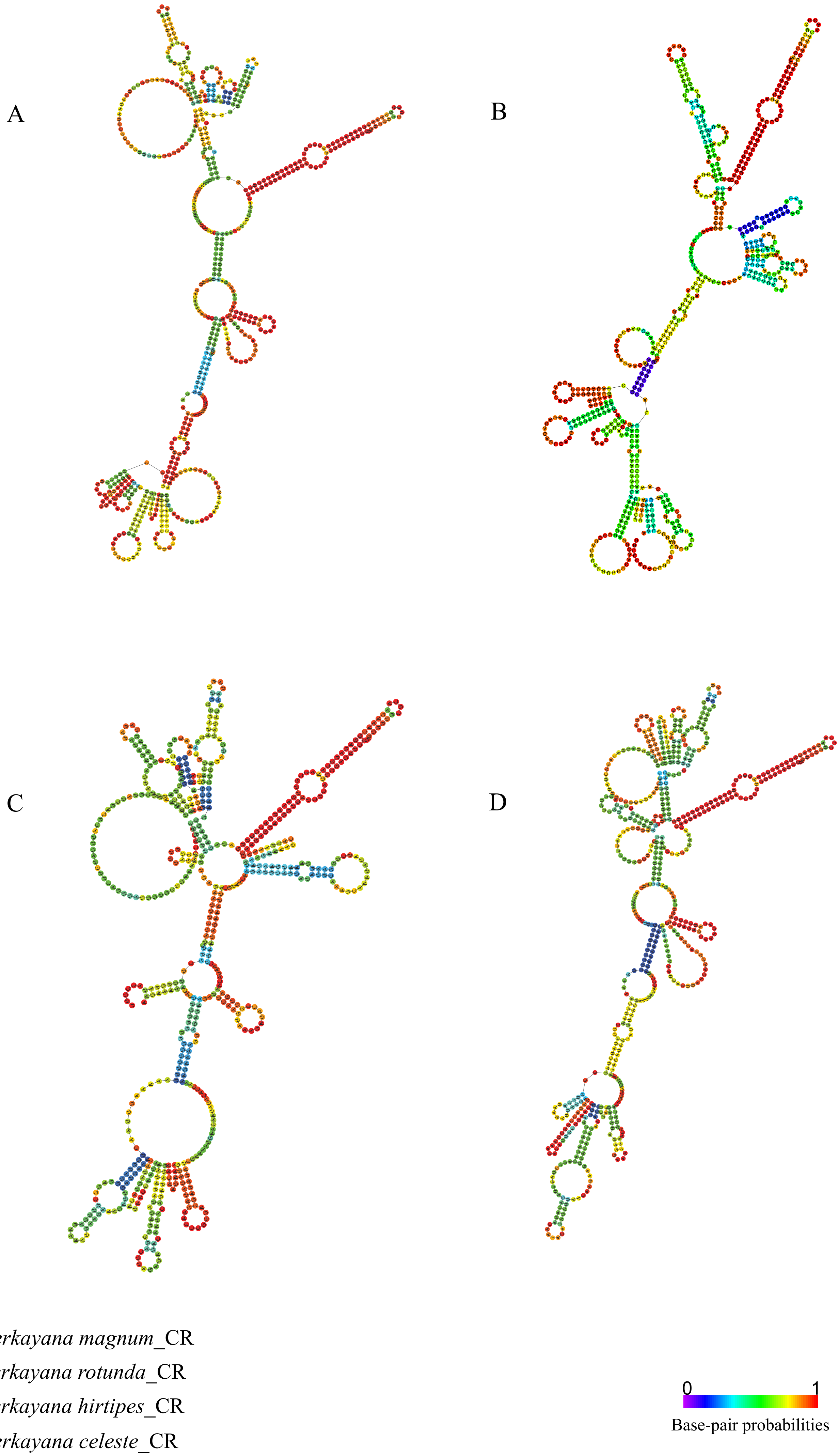

A: *Tuerkayana magnum*_CR
B: *Tuerkayana rotunda*_CR
C: *Tuerkayana hirtipes*_CR
D: *Tuerkayana celeste*_CR
