# Supplementary figures and images for: Molecular Phylogeny and Evolution of the Tuerkayana (Decapoda: Brachyura: Gecarcinidae) Genus Based on Whole Mitochondrial Genome Sequences
Source: Biology (Basel). 2023 Jul 8;12(7):974. doi: 10.3390/biology12070974 (PMC10376310; doi:10.3390/biology12070974)

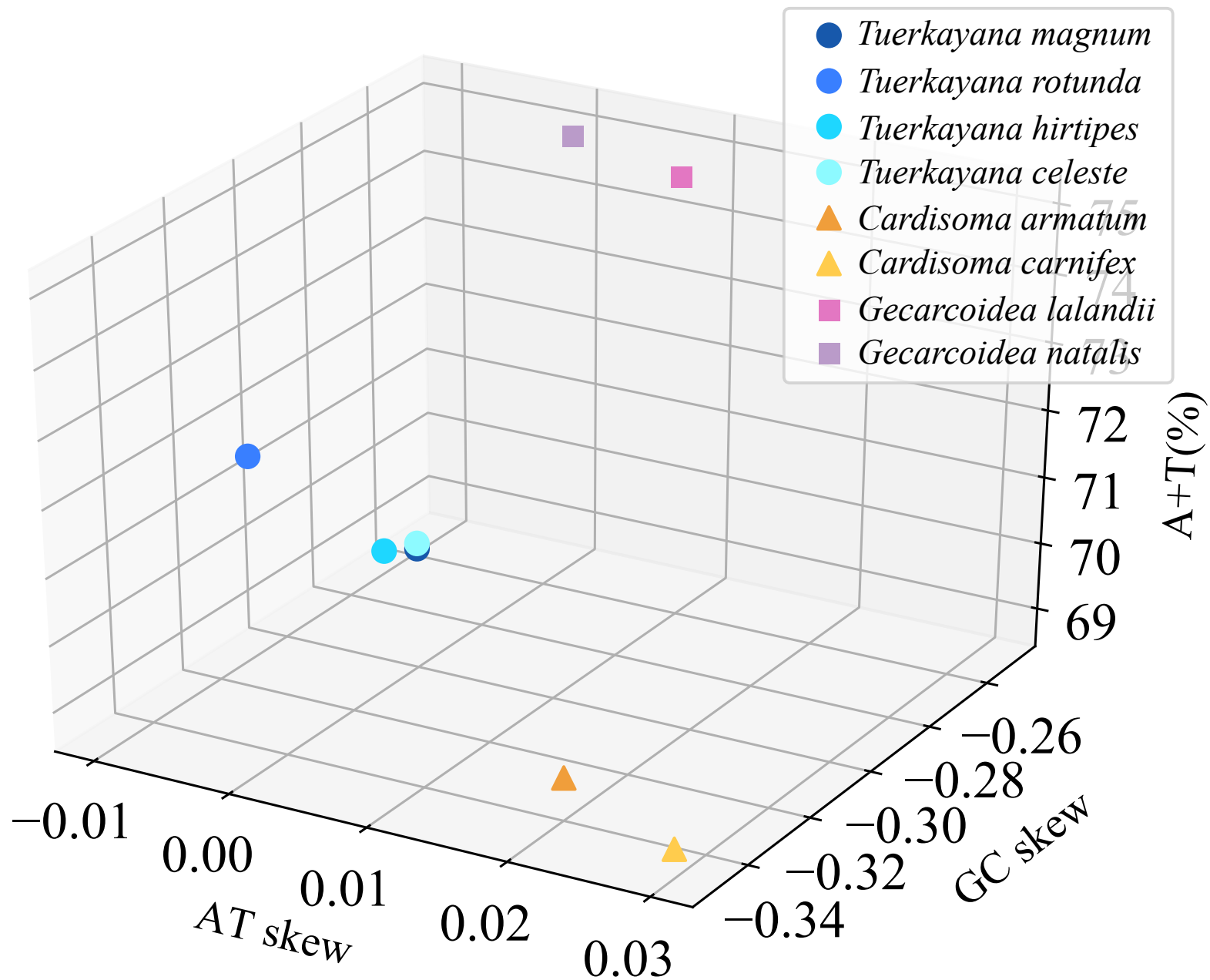

Supplement: Supplementary file 1 [file biology-12-00974-s001.zip › Figure S1.pdf]

tmA

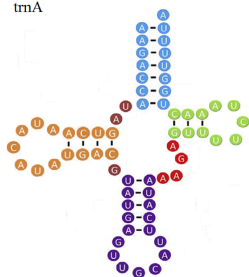

tmC

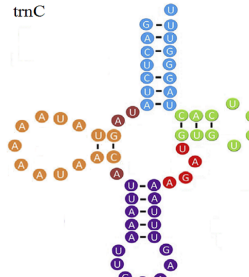

tmD

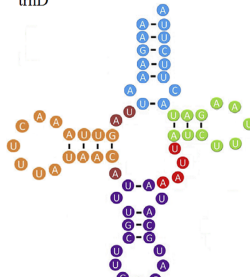

tmE

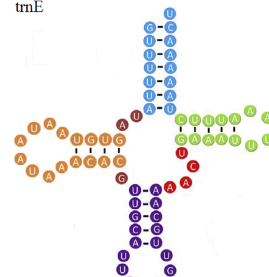

tmF

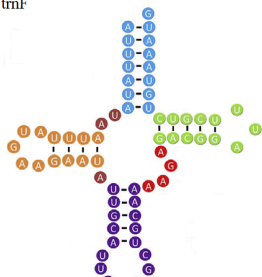

tmG

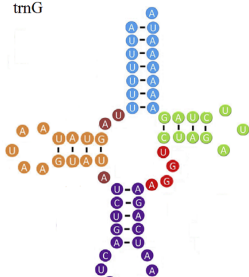

tmH

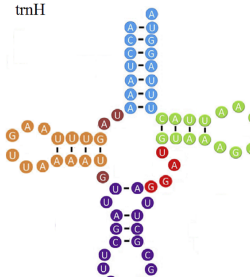

tmI

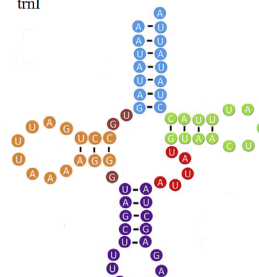

tmK

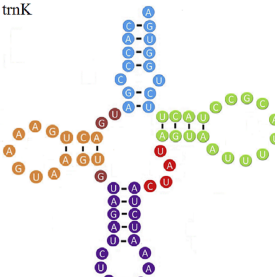

tmL1

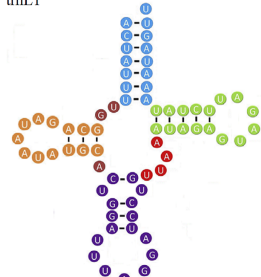

tmL2

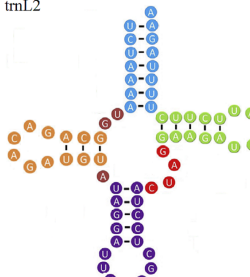

tmM

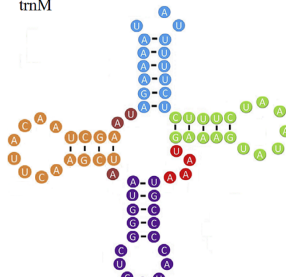

tmN

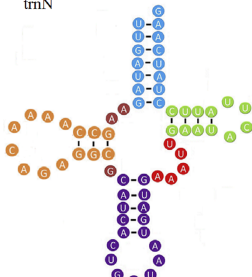

tmP

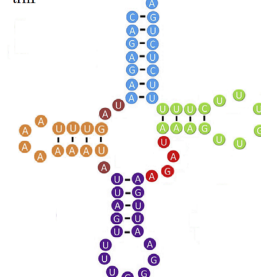

tmQ

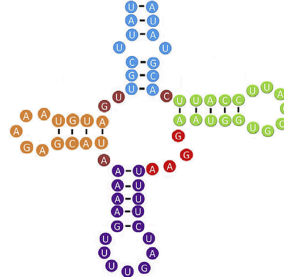

tmR

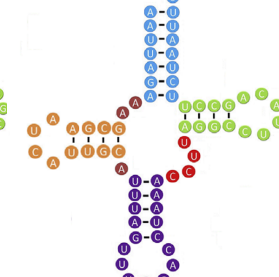

tmS1

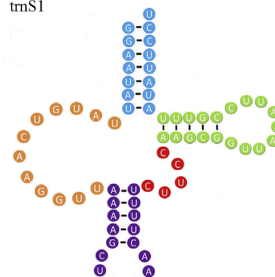

tmS2

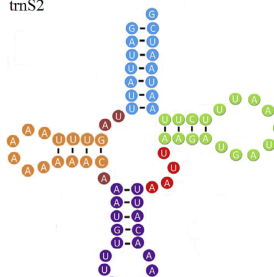

tmT

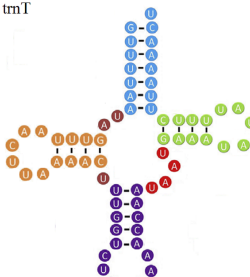

tmV

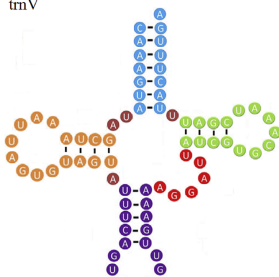

tmW

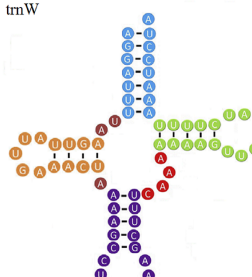

tmY

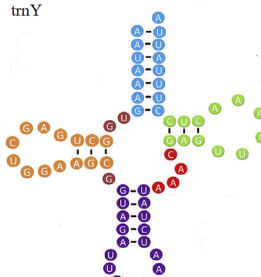

tmF

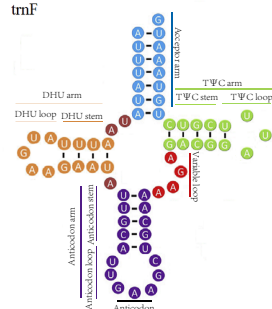

Supplement: Supplementary file 1 [file biology-12-00974-s001.zip › Figure S2.pdf]

*Tuerkayana rotunda*

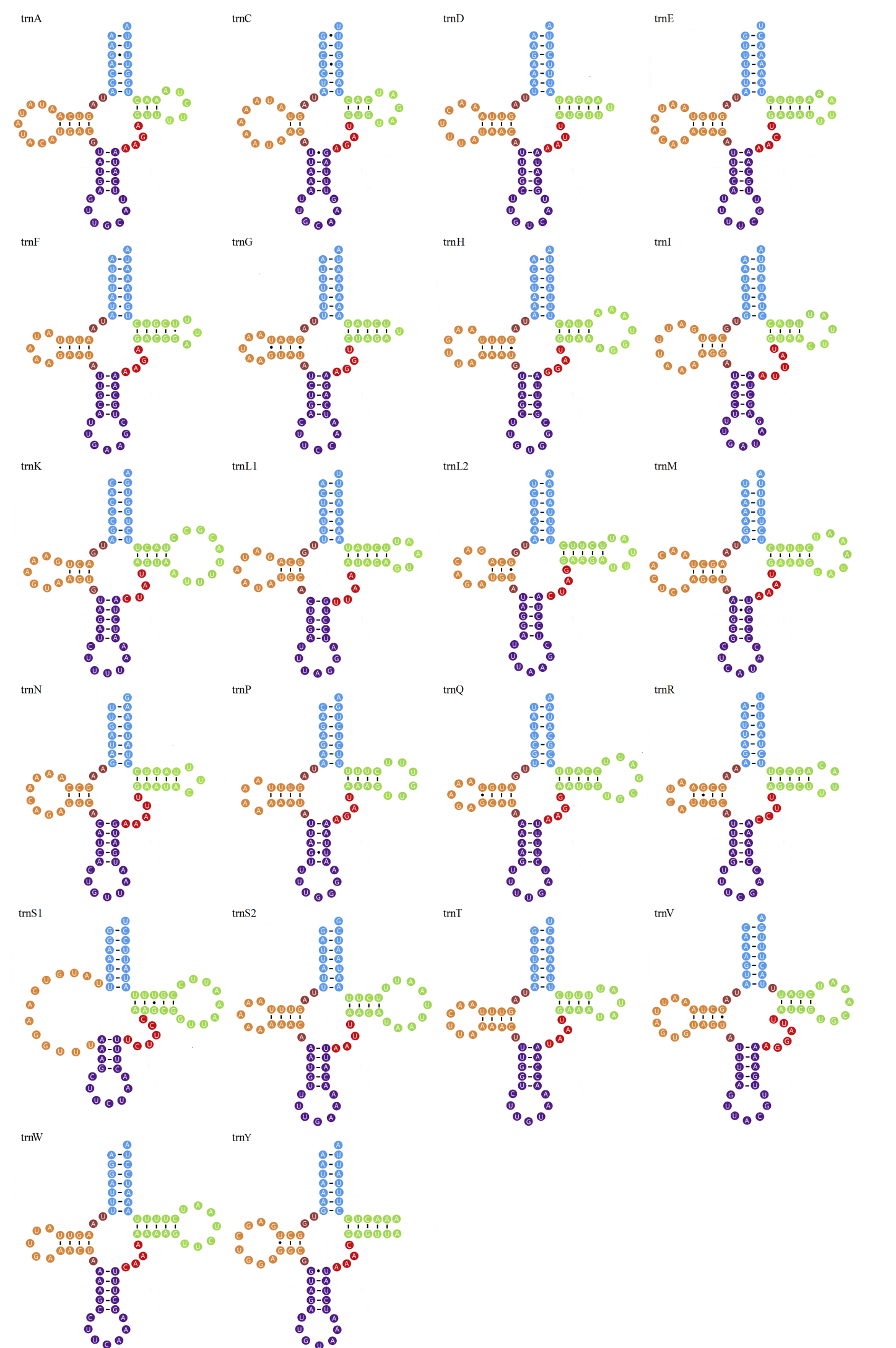

*Tuerkayana hirtipes*

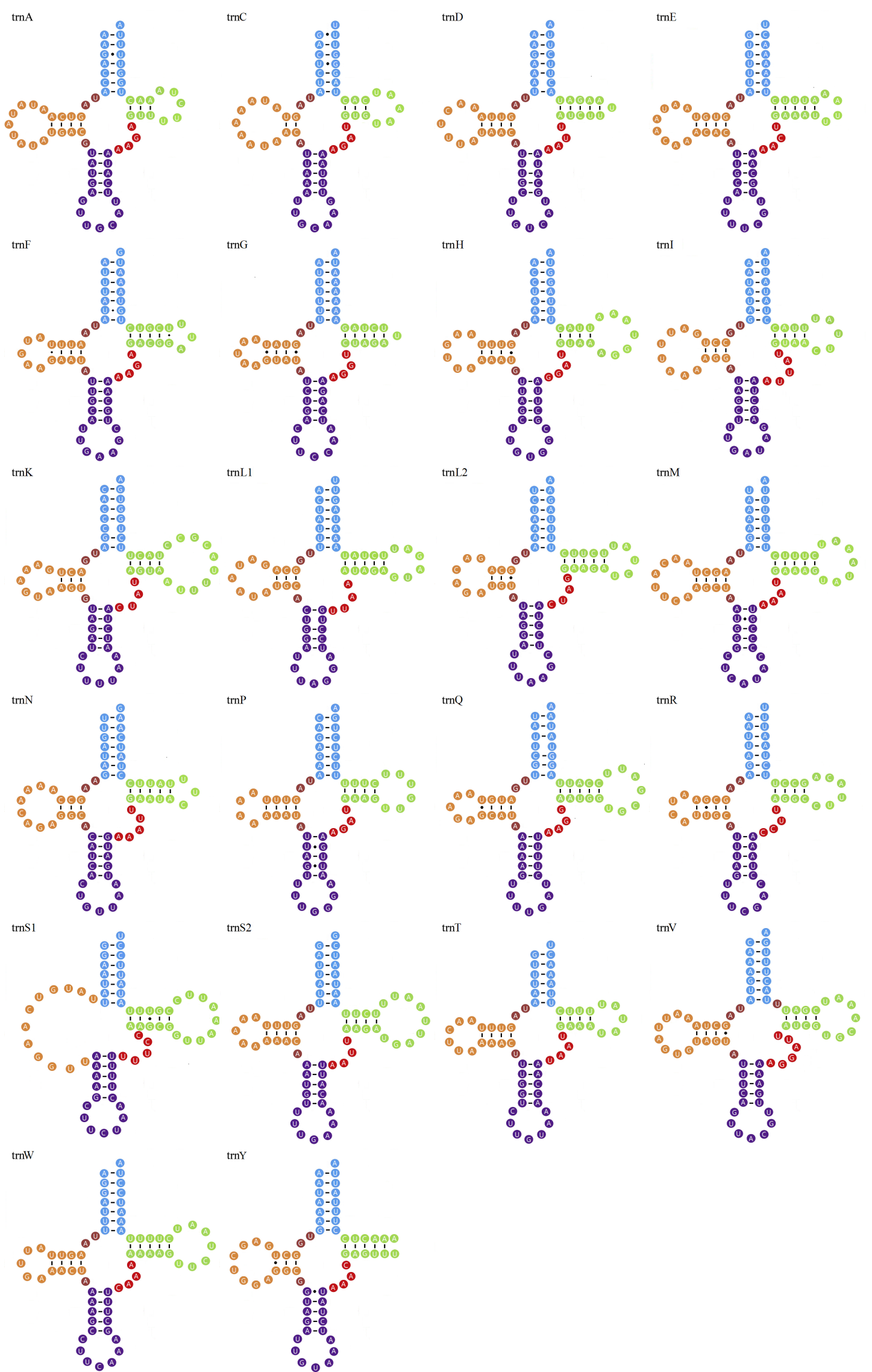

*Tuerkayana celeste*

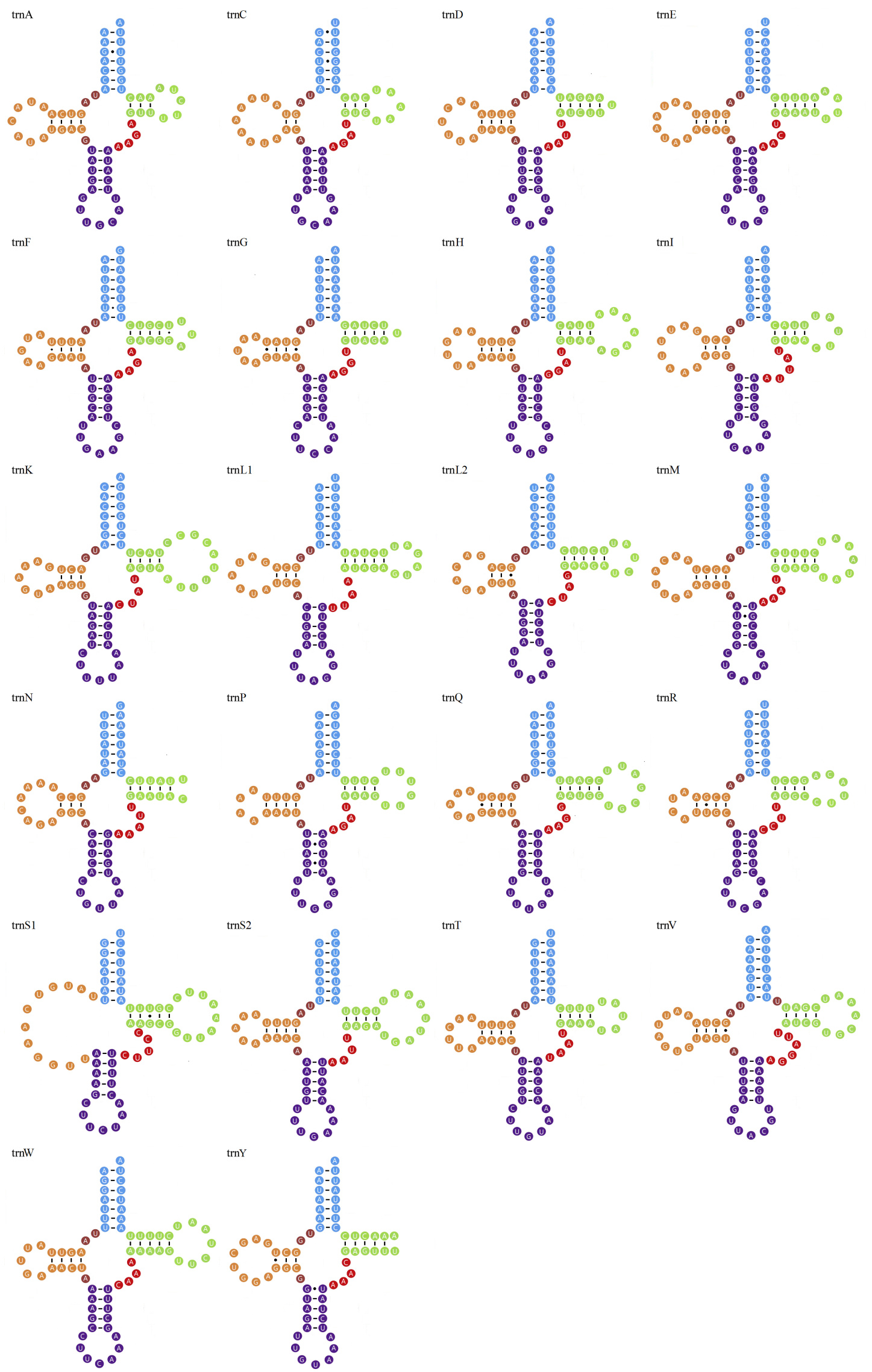

Supplement: Supplementary file 1 [file biology-12-00974-s001.zip › Figure S3.pdf]

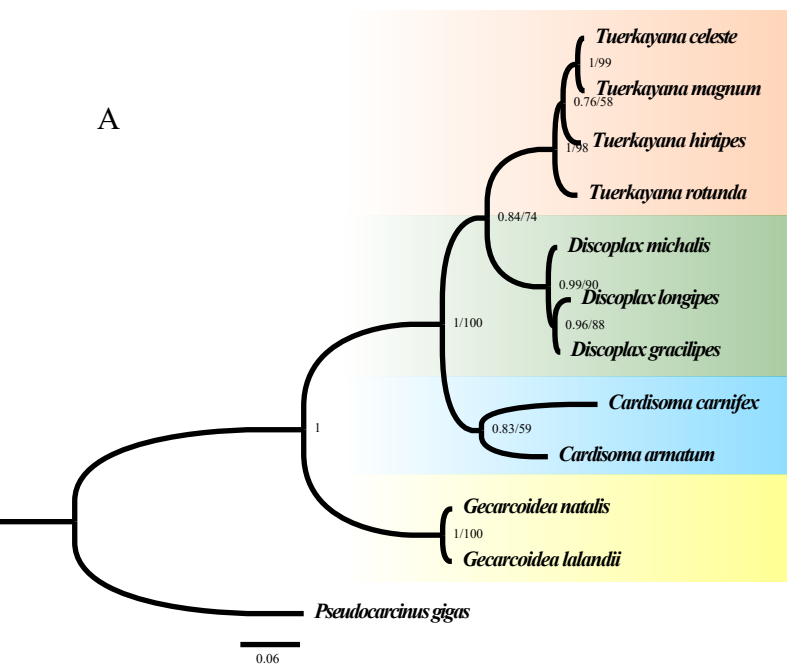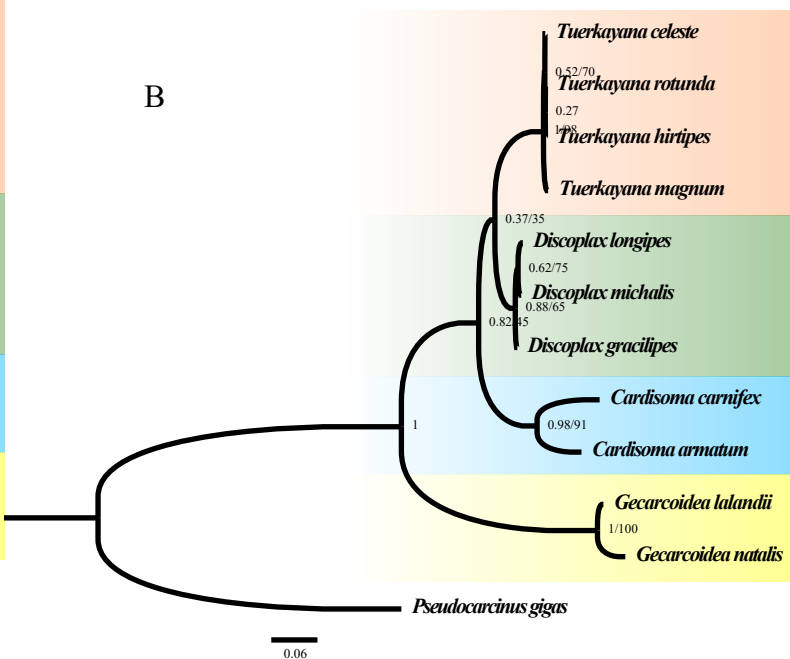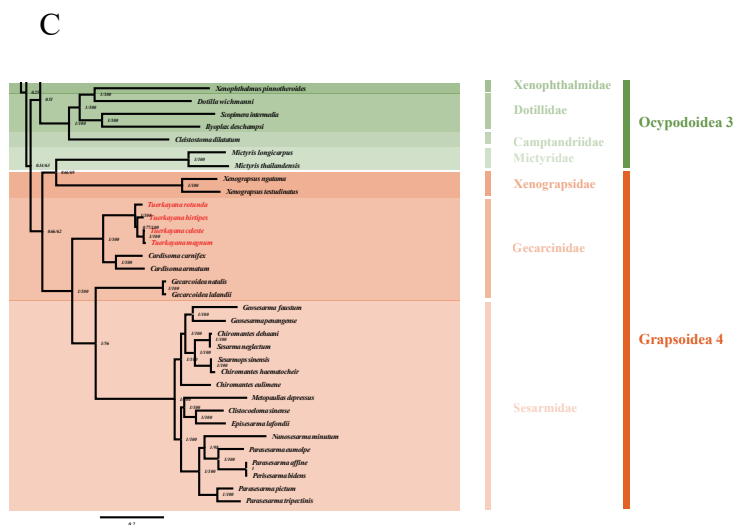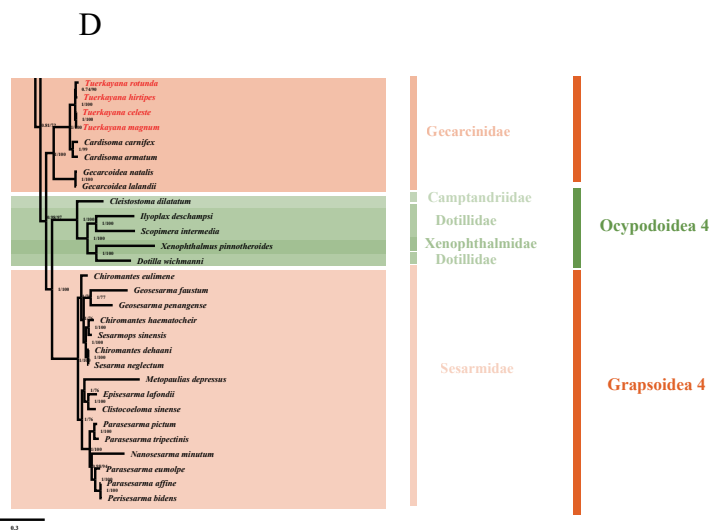

Supplement: Supplementary file 1 [file biology-12-00974-s001.zip › Figure S6.pdf]

*ATP6*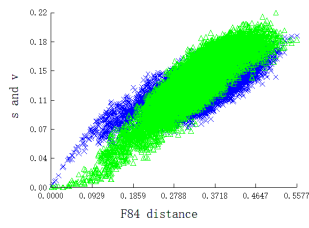*ATP8*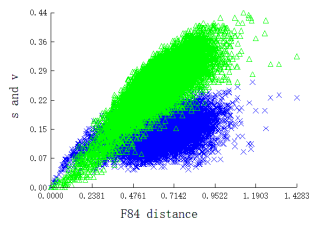*COX1*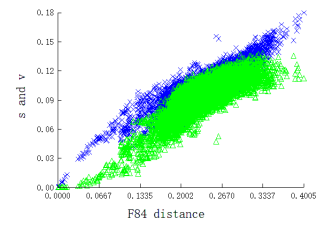*COX2*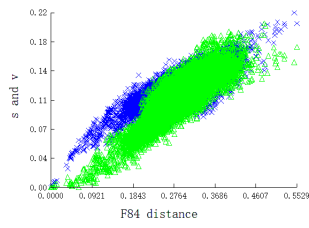*COX3*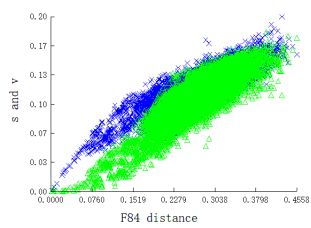*CYTB*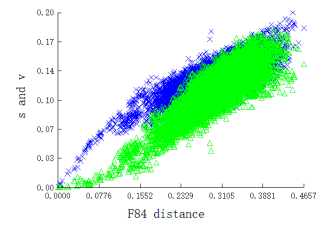*ND1*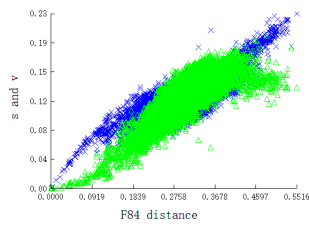*ND2*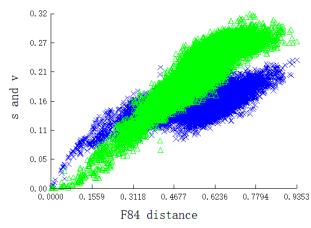*ND3*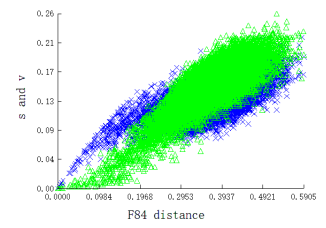*ND4*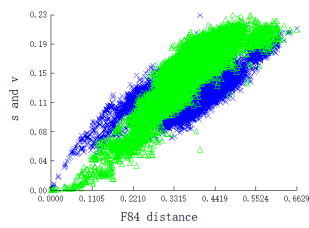*ND4L*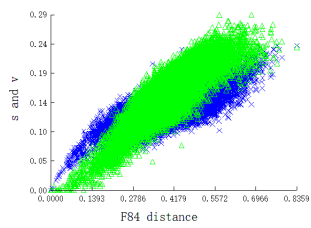*ND5*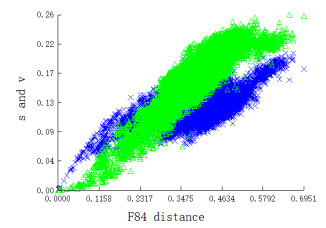*ND6*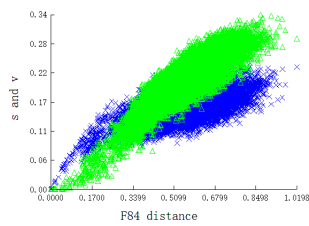

Supplement: Supplementary file 1 [file biology-12-00974-s001.zip › Figure S7.pdf]

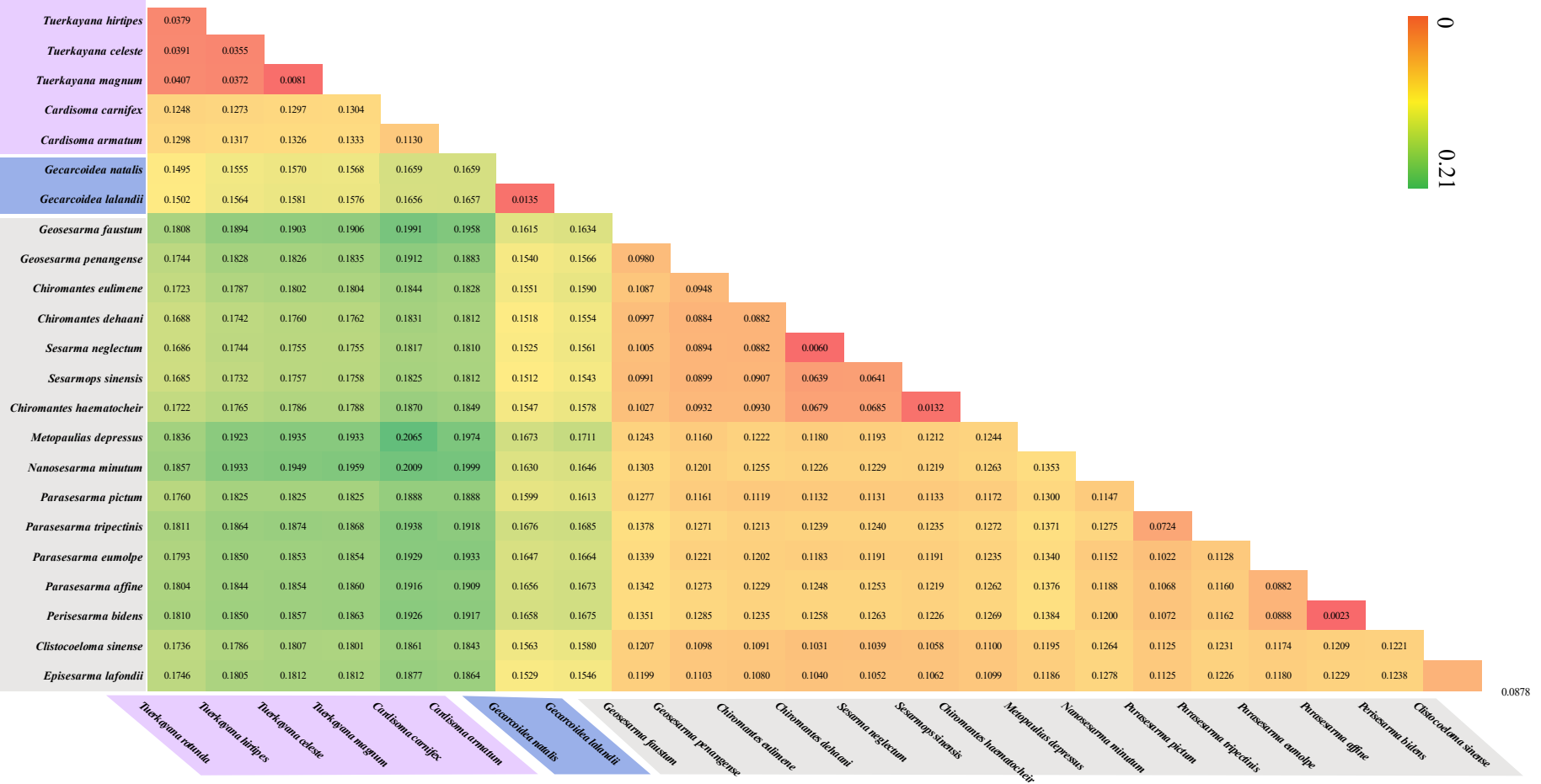

Supplement: Supplementary file 1 [file biology-12-00974-s001.zip › Figure S9.pdf]
