# Supplementary material for: Molecular Phylogeny and Evolution of the Tuerkayana (Decapoda: Brachyura: Gecarcinidae) Genus Based on Whole Mitochondrial Genome Sequences
Source: Biology (Basel). 2023 Jul 8;12(7):974. doi: 10.3390/biology12070974 (PMC10376310; doi:10.3390/biology12070974)
Supplement: Supplementary file 1 [file Figure S8.pdf › Figure S8.pdf]

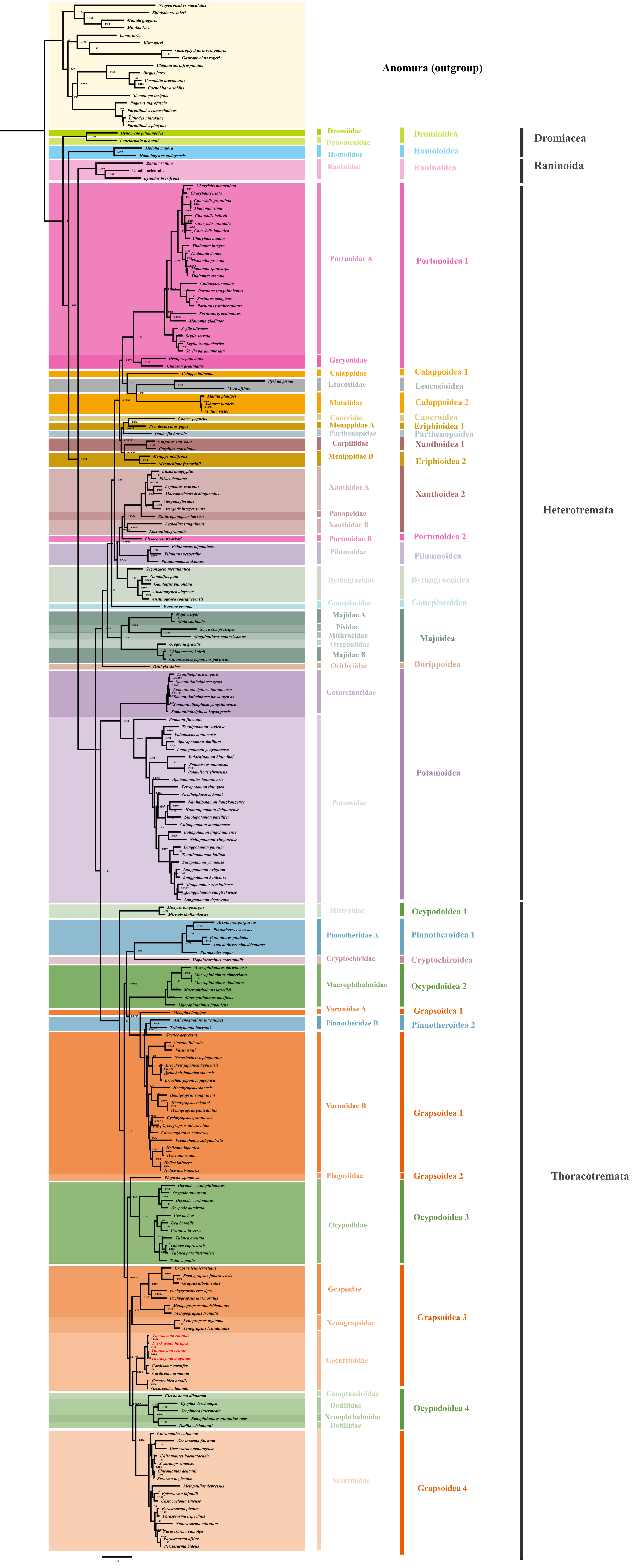

Anomura (outgroup)
Dromiidae
Dynomenidae
Homolidae
Raninidae
Portunidae A
Geryonidae
Calappidae
Leucosiidae
Matutidae
Cancridae
Menippidae A
Parthenopidae
Carpiliidae
Menippidae B
Xanthidae A
Panopeidae
Xanthidae B
Portunidae B
Pilumnidae
Bythograeidae
Goneplacidae
Majidae A
Pisidae
Mithracidae
Oregoniidae
Majidae B
Orithyiidae
Gecarcinucidae
Potamidae
Mictyridae
Pinnotheridae A
Cryptochiridae
Macrophthalmidae
Varunidae A
Pinnotheridae B
Varunidae B
Plagusiidae
Ocypodidae
Grapsidae
Xenograpsidae
Gecarcinidae
Camptandriidae
Dotillidae
Xenophthalmidae
Dotillidae
Sesarmidae
Dromioidea
Homoloidea
Raninoidea
Portunoidea 1
Calappoidea 1
Leucosioidea
Calappoidea 2
Cancroidea
Eriphioidea 1
Parthenopoidea
Xanthoidea 1
Eriphioidea 2
Xanthoidea 2
Portunoidea 2
Pilumnoidea
Bythograeoidea
Goneplacoidea
Majoidea
Dorippoidea
Potamoidea
Ocypodoidea 1
Pinnotheroidea 1
Cryptochiroidea
Ocypodoidea 2
Grapsoidea 1
Pinnotheroidea 2
Grapsoidea 1
Grapsoidea 2
Ocypodoidea 3
Grapsoidea 3
Ocypodoidea 4
Grapsoidea 4
Dromiacea
Raninoida
Heterotremata
Thoracotremata
0.1
